# Disease modeling of pulmonary fibrosis using human pluripotent stem cell-derived alveolar organoids

**DOI:** 10.1016/j.stemcr.2021.10.015

**Published:** 2021-11-18

**Authors:** Takahiro Suezawa, Shuhei Kanagaki, Keita Moriguchi, Atsushi Masui, Kazuhisa Nakao, Masayasu Toyomoto, Koji Tamai, Ryuta Mikawa, Toyohiro Hirai, Koji Murakami, Masatoshi Hagiwara, Shimpei Gotoh

**Affiliations:** 1 Department of Drug Discovery for Lung Diseases, Graduate School of Medicine, Kyoto University, Kyoto, Japan; 2 Watarase Research Center, Kyorin Pharmaceutical Co., Ltd., Shimotsuga-gun, Tochigi, Japan; 3 Department of Anatomy and Developmental Biology, Graduate School of Medicine, Kyoto University, Kyoto, Japan; 4 Department of Respiratory Medicine, Graduate School of Medicine, Kyoto University, Kyoto, Japan

**Keywords:** pulmonary fibrosis, pluripotent stem cells, epithelial-mesenchymal interaction, ALK5, integrin αVβ6

## Abstract

Although alveolar epithelial cells play a critical role in the pathogenesis of pulmonary fibrosis, few practical *in vitro* models exist to study them. Here, we established a novel *in vitro* pulmonary fibrosis model using alveolar organoids consisting of human pluripotent stem cell-derived alveolar epithelial cells and primary human lung fibroblasts. In this human model, bleomycin treatment induced phenotypes such as epithelial cell-mediated fibroblast activation, cellular senescence, and presence of alveolar epithelial cells in abnormal differentiation states. Chemical screening performed to target these abnormalities showed that inhibition of ALK5 or blocking of integrin αVβ6 ameliorated the fibrogenic changes in the alveolar organoids. Furthermore, organoid contraction and extracellular matrix accumulation in the model recapitulated the pathological changes observed in pulmonary fibrosis. This human model may therefore accelerate the development of highly effective therapeutic agents for otherwise incurable pulmonary fibrosis by targeting alveolar epithelial cells and epithelial-mesenchymal interactions.

## Highlights

• Human pluripotent stem cell-based *in vitro* pulmonary fibrosis model was established
• Bleomycin-treated alveolar organoids showed epithelium-dependent contraction
• Abnormal differentiation state and cellular senescence in AT2 cells were mimicked
• Inhibition of TGFβ signaling ameliorated the fibrogenic changes of the disease model

## Graphical abstract

**Figure:**
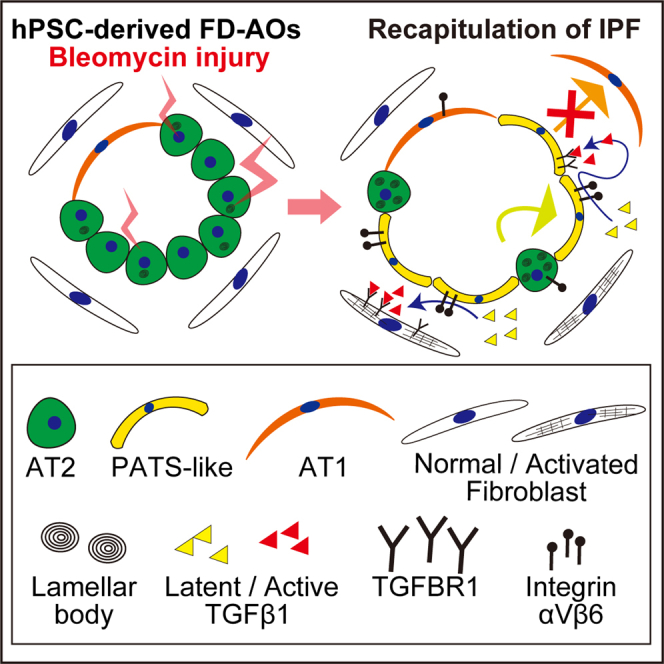

## Teaser

Gotoh and colleagues established a human pluripotent stem cell-based *in vitro* pulmonary fibrosis model. The human model recapitulates pathogenic changes of pulmonary fibrosis, such as tissue contraction with epithelial cell-mediated fibroblast activation, cellular senescence, and abnormal differentiation states of alveolar epithelial cells. These findings could accelerate drug discovery for pulmonary fibrosis, targeting alveolar epithelial cells and epithelial-mesenchymal interactions.

## Introduction

Idiopathic pulmonary fibrosis (IPF) is a chronic, progressive interstitial lung disease that is more likely to occur in the elderly. Mutations in genes related to telomere homeostasis, such as *TERT*, *TERC*, *PARN*, and *RTEL1*, are associated with an increased risk of sporadic IPF (Lederer and Martinez, 2018). Therefore, aging, or at least cellular senescence, is believed to be involved in the onset of IPF. The pathogenesis of IPF is fundamentally based on initial alveolar epithelial injury followed by fibroblast activation (Katzen and Beers, 2020). Pirfenidone and nintedanib, the only two drugs for IPF approved by the US Food and Drug Administration, mainly target fibroblasts involved in the late stage of IPF (Spagnolo et al., 2020). Thus, there still remains a need for new therapeutic drugs targeting epithelial cells involved in the early stage of IPF.

Alveolar type 2 (AT2) cells are major tissue stem cells capable of secreting pulmonary surfactant in alveoli (Barkauskas et al., 2013). They can self-renew and differentiate into alveolar type 1 (AT1) cells, which are responsible for the gas exchange in the entire human body. Several reports suggest that AT2 cells play critical roles in pulmonary fibrosis. Mutations in surfactant-related genes expressed in AT2 cells are common in familial interstitial pneumonia (Katzen and Beers, 2020). There are several reports that mice with specific damage to AT2 cells develop pulmonary fibrosis (Sisson et al., 2010; Yao et al., 2021), which supports the hypothesis that the interactions between alveolar epithelial cells and fibroblasts may contribute to fibrogenesis (Katzen and Beers, 2020).

To study these interactions, *in vitro* models such as co-cultures and organoids have been pursued (Agarwal et al., 2001; Tan et al., 2019). However, these models have shown little direct evidence of the epithelial-mesenchymal interactions involved in fibroblast activation or epithelial cell damage, due to the following three limitations. First, there are few reports using functional AT2 cells as tissue stem cells that can maintain surfactant homeostasis. Instead, most studies have used airway epithelial cells because of the difficulties of long-term culture of alveolar epithelial cells. Second, the low throughput and reproducibility of existing organoid models hamper drug screening. Third, most models use mouse cells, and there are species-specific differences between humans and mice in the distal lung (Basil et al., 2020). Considering these factors, a human *in vitro* pulmonary fibrosis model that can maintain alveolar epithelial cell function and allow fibroblast crosstalk is required, as it can allow for the screening of potential targets with sufficient throughput to be feasible for drug discovery.

We previously reported a method of generating fibroblast-dependent alveolar organoids (FD-AOs) from human pluripotent stem cells (hPSCs) and primary human fetal lung fibroblasts (HFLFs) (Gotoh et al., 2014; Yamamoto et al., 2017). These organoids could be expanded in the long term, and consisted of multi-lineage epithelial cells derived from hPSCs and fibroblasts. Our previous reports on AT2 cell dysfunction associated with amiodarone (AMD)-induced and Hermansky-Pudlak syndrome type 2-related pulmonary fibrosis lacked a demonstration of the phenotypes of pulmonary fibrosis or activated fibroblasts associated with epithelial cell injury (Kanagaki et al., 2021b; Korogi et al., 2019). Here, we developed a novel pulmonary fibrosis model *in vitro* using surfactant protein C-positive (SPC^+^) cell-derived FD-AOs upon bleomycin (BLM) treatment. The results from our study suggest that FD-AOs could recapitulate the interactions of human alveolar epithelial cells and fibroblasts in pulmonary fibrosis *in vitro* and be useful for screening therapeutic agents to treat IPF.

## Results

### BLM treatment induces contraction of FD-AOs in an epithelial cell-dependent manner

**Figure 1:**
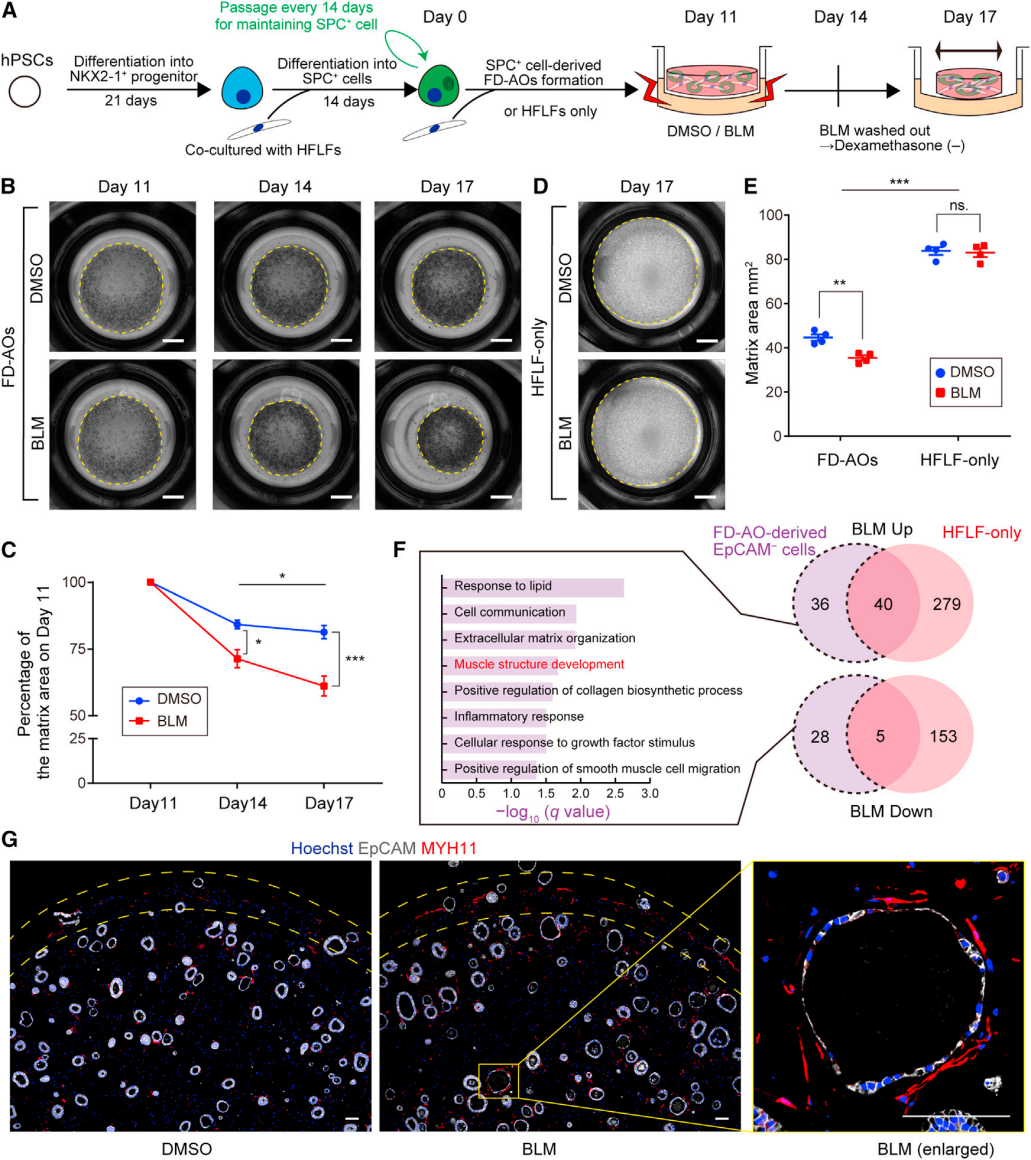
Contraction of FD-AO matrices in response to BLM treatment is dependent on epithelial cells (A) Experimental scheme. (B and C) Whole-well imaging and quantification of the area of cultivation matrices of FD-AOs on days 11, 14, and 17. Scale bars, 2 mm. (D) Whole-well imaging of the matrices of HFLF-only culture. Scale bars, 2 mm. (E) Quantification of the matrix area of FD-AOs and HFLF-only culture. (F) GO analysis of DEGs specific to EpCAM^−^ cells derived from BLM-treated FD-AOs. DESeq2 was used to identify the DEGs for each condition, and the threshold was set to the adjusted p < 0.05 (n = 3). (G) IFA of cultivation matrix of FD-AOs. Gray, EpCAM; red, MYH11; blue, nuclei (Hoechst). Scale bars, 100 μm. Data are presented as mean ± SEM (n = 4 independent experiments). Two-way ANOVA with Sidak’s multiple comparisons test: ^∗^p < 0.05, ^∗∗^p < 0.01, ^∗∗∗^p < 0.001; ns, not significant.

BLM, which exhibits anticancer effects through DNA double-strand breaks, causes pulmonary fibrosis as a side effect and is most commonly used as an experimental pulmonary fibrosis inducer in *in vivo* mouse models (Moeller et al., 2008). Therefore, we investigated whether treating FD-AOs with BLM could establish a model of pulmonary fibrosis. First of all, we optimized the concentration of BLM by the expression level of *CDKN1A*, an indicator of DNA damage (Cazzalini et al., 2010). The expression of *CDKN1A* was upregulated in a BLM concentration-dependent manner and persisted for 3 days after BLM removal (Figures 1A and S1A).

One of the phenotypes that indicates pulmonary fibrosis is contraction of the lung tissue, involving cell contraction of activated fibroblasts (Tomasek et al., 2002). Three-dimensional collagen gels have been widely used for analyzing the cell contraction in the fibroblast culture models (Montesano and Orci, 1988; Wilkinson et al., 2017). Since FD-AOs were encapsulated in Matrigel, which is mainly composed of laminin, collagen, and a reduced amount of growth factors, we investigated whether fibroblast activation could cause contraction of the cultivation matrix. Treating FD-AOs with BLM caused shrinkage of the matrix in a time-dependent manner (Figures 1B and 1C). To evaluate whether epithelial cells contributed to this shrinkage, we conducted control experiments on three-dimensional cultured HFLFs without epithelial cells. Interestingly, BLM-induced shrinkage occurred more strongly in the FD-AOs than in HFLF-only matrices (Figures 1D and 1E). This shrinkage was observed regardless of the clone of hPSC-derived alveolar epithelial cells or lung fibroblasts (Figures S1B–S1E). However, the presence of epithelial cells caused the cultivation matrices to shrink even without BLM.

Next, we evaluated the transcriptomes of fibroblasts under each condition (Table S1). Principal component analysis showed that the presence or absence of epithelial cells had a greater effect on overall gene expression in fibroblasts than treatment with BLM (Figure S2A), which was consistent with the matrix shrinkage. In the dimethyl sulfoxide (DMSO)-treated condition, the gene that was most strongly induced by the presence of epithelial cells was *PTCH1*, one of the major hedgehog receptors (Figure S2B). Hedgehog signaling-related genes such as *HHIP* and *GLI1* were also included in the top ten upregulated genes. Further, gene set enrichment analysis (GSEA) indicated the activation of hedgehog signaling (nominal p value = 0.0018, false discovery rate [FDR] q value = 0.068) (Figure S2C). These findings were consistent with a recent report by Zepp et al. (2021) showing that hedgehog signaling from AT1 cells was required for the induction of secondary crest myofibroblasts (SCMFs), which are described as highly contractile fibroblasts that are present during lung development. Because of the presence of AT1-like cells in our organoids (Kanagaki et al., 2021a), we assessed whether SCMF lineage markers varied depending on the presence or absence of epithelial cells. The SCMF low-expression markers, *WNT2* and *TCF21*, were decreased while the SCMF high-expression marker, *STC1*, was upregulated in the presence of epithelial cells (Figure S2D). Furthermore, the most common hedgehog ligand, *SHH*, was expressed specifically in EpCAM^+^ cells in FD-AOs (Figure S2E), and the addition of cyclopamine, which inhibits hedgehog signaling (Chen et al., 2002), suppressed epithelial cell-dependent matrix shrinkage (Figures S2F and S2G). These results suggest that fibroblasts of FD-AOs acquire the contractile characteristics similar to those of SCMFs via hedgehog signals from epithelial cells during matrix shrinkage under the DMSO-treated condition.

Differentially expressed genes (DEGs) induced by BLM treatment in fibroblasts of FD-AOs and in HFLF-only cultures partially overlapped (Figure 1F). However, some DEGs were specifically changed in each condition. We analyzed the DEGs induced by BLM treatment only in the presence of epithelial cells (Table S1). Gene ontology (GO) enrichment analysis of biological processes showed that the pathways associated with “Response to lipid” and “Cell communication” were significantly enriched (Figure 1F). GO results indicated probable interactions between fibroblasts and epithelial cells, especially AT2 cells having lamellar bodies, which are surfactant lipid-storing organelles. Other enrichments included “Extracellular matrix organization” and “Muscle structure development.” *MYH11*, which encodes myosin heavy chain 11, has been reported to be upregulated in the lung fibroblasts of IPF and BLM model mice (Reyfman et al., 2019; Xie et al., 2018). *MYH11* was upregulated by BLM only in the presence of epithelial cells (Table S1), and immunofluorescence analysis (IFA) showed that MYH11^+^ fibroblasts appeared at the surrounding edge of the cultivation matrix and was preferentially observed along with spheroids in BLM-treated FD-AOs (Figure 1G). These results suggest that epithelial cells contribute to the BLM-induced fibroblast activation.

### BLM treatment induces morphological change in epithelial cells in FD-AOs

**Figure 2:**
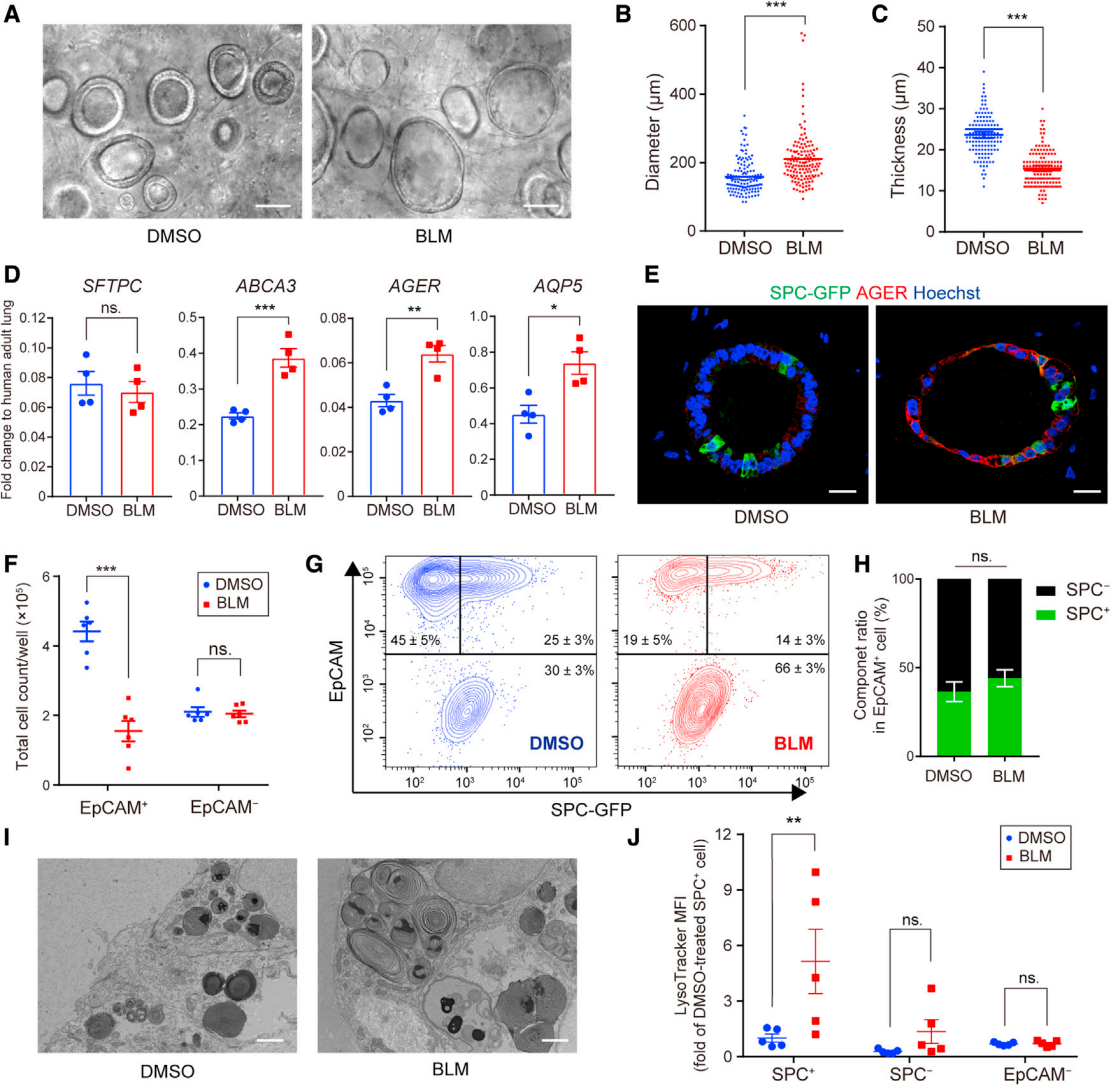
BLM induces morphological change in epithelial cells in FD-AOs (A) Live-cell imaging of epithelial cells in BLM-treated FD-AOs. Scale bars, 100 μm. (B and C) Quantification of diameter and thickness of alveolar spheroids. Data are presented as mean ± SEM (n = 140 spheroids from 7 independent experiments). Unpaired two-tailed Student’s t test: ^∗∗∗^p < 0.001. (D) Expression levels of AT2 (*SFTPC*, *ABCA3*) and AT1 (*AQP5*, *AGER*) markers in FD-AOs evaluated by qRT-PCR. Gene expression in the normalizer (adult lung RNA control) was set at 1. Data are presented as mean ± SEM (n = 4). Unpaired two-tailed Student’s t test: ^∗^p < 0.05, ^∗∗^p < 0.01, ^∗∗∗^p < 0.001; ns, not significant. (E) IFA of FD-AOs. Green, SPC-GFP; red, AGER; blue, nuclei (Hoechst). Scale bars, 20 μm. (F) Total number of EpCAM^+^ or EpCAM^−^ cells in a well. All dissociated cells were counted and multiplied by the ratio of EpCAM^+^ to EpCAM^−^ cells as quantified by FCM. Data are mean ± SEM (n = 6 independent experiments). Two-way ANOVA with Sidak’s multiple comparisons test: ^∗∗∗^p < 0.001; ns, not significant. (G) FCM of DMSO- and BLM-treated FD-AOs. (H) Analyses of cell component ratio of EpCAM^+^ cells in FD-AOs using FCM. Data are presented as mean ± SEM (n = 5 independent experiments). Unpaired two-tailed Student’s t test: ns, not significant. (I) Transmission electron microscopy images of BLM-treated FD-AOs. Scale bars, 2 μm. (J) Mean fluorescence intensity of LysoTracker in FCM. Data are presented as mean ± SEM (n = 5 independent experiments). Two-way ANOVA with Sidak’s multiple comparisons test: ^∗∗^p < 0.01; ns, not significant.

We investigated whether BLM treatment caused morphological changes in epithelial cells in FD-AOs (Figure 2A). BLM treatment increased the diameter and decreased the thickness of the alveolar spheroids (Figures 2B and 2C). Since cuboidal AT2 cells can differentiate into thin and flat-shaped AT1 cells, we hypothesized that BLM affected the stemness of AT2 cells. We then evaluated the gene expression of AT1 and AT2 cell-specific markers in FD-AOs (Figure 2D). Among AT2-specific markers, BLM treatment did not significantly change the expression of *SFTPC* but did upregulate the expression of *ABCA3*. AT1-specific markers *AQP5* and *AGER* were significantly upregulated in BLM-treated FD-AOs. Furthermore, IFA showed an enhanced expression of AGER in BLM-treated FD-AOs (Figure 2E).

We counted the number of EpCAM^+^ or EpCAM^−^ cells in BLM-treated FD-AOs to investigate whether the elevated epithelial cell markers were due to an increase in the number of epithelial cells. BLM treatment decreased the number of EpCAM^+^ cells but did not change the number of EpCAM^−^ cells (Figure 2F). Consistent with mRNA expression, there was no significant difference in the positive rate of SPC-GFP in EpCAM^+^ cells (Figures 2G and 2H). These results suggest that, upon BLM treatment, the spheroid-growing capacity of AT2 cells was impaired but their differentiation status was maintained or upregulated.

We previously reported a method to culture hPSC-derived alveolar epithelial cells without feeder fibroblasts: fibroblast-free alveolar organoids (FF-AOs). We treated FF-AOs with BLM to evaluate its direct effect (Figure S3A). As with FD-AOs, the number of viable epithelial cells in FF-AOs was reduced by BLM treatment (Figure S3B). However, BLM treatment in FF-AOs decreased the diameter of alveolar spheroids and did not increase AT1-specific markers other than *CAV1* (Figures S3C–S3E). These results suggest that BLM directly disrupts alveolar epithelial cells, but the differentiation program to AT1 cells does not work in the absence of fibroblasts.

Next, since AT2 cells store surfactant lipid in lamellar bodies, we examined the ultrastructure of AT2 cells in BLM-treated FD-AOs using a transmission electron microscope. Hypertrophic lamellar bodies were noted in BLM-treated FD-AOs (Figure 2I). We previously reported that AMD treatment induced hypertrophic lamellar bodies in alveolar epithelial cells and that LysoTracker, which binds to acidic phospholipids, stained not only AT2 cells but also the other epithelial cells and fibroblasts in AMD-treated FD-AOs (Kanagaki et al., 2021b; Yamamoto et al., 2017). However, in the present study, LysoTracker staining demonstrated that BLM treatment induced phospholipid accumulation specifically in SPC^+^ cells in FD-AOs (Figure 2J). This preference for SPC^+^ cells was also observed in FF-AOs (Figures S3F–S3H).

### BLM-treated FD-AOs involve AT2-AT1 intermediate-state cells

**Figure 3:**
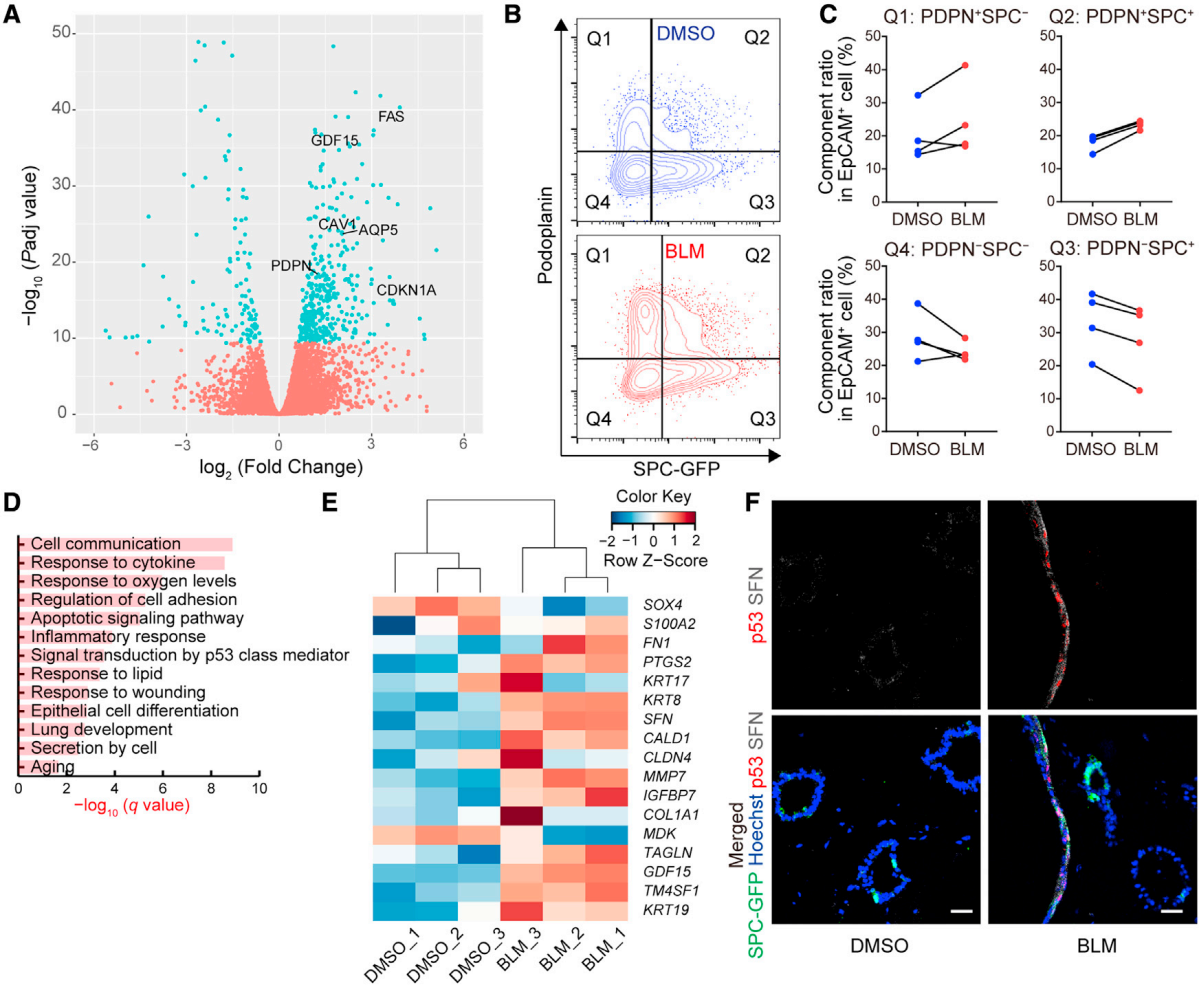
BLM-treated epithelial cells involve AT2-AT1 intermediate-state cells in FD-AOs (A) Volcano plot obtained from the DESeq2 analysis of SPC-GFP^+^ cell with or without BLM treatment (n = 3 independent experiments). SPC-GFP^+^ cells were separated from FD-AOs. (B and C) FCM analysis of PDPN and SPC-GFP in EpCAM^+^ gated cells (n = 4 independent experiments). (D) GO analysis using the top 500 DEGs upon BLM treatment in SPC^+^ cells. (E) Heatmap indicating *Z* scores of the PATS markers. The log_2_ (TPM values) of SPC^+^ cells were used to calculate *Z* scores (n = 3 independent experiments). (F) IFA of FD-AOs. Green, GFP-SPC; red, p53; gray, SFN; blue, nuclei (Hoechst). Scale bars, 25 μm.

We performed RNA sequencing (RNA-seq) of isolated SPC-GFP^+^ cells from BLM-treated FD-AOs (Table S2). Although SPC^+^ cells are mainly AT2 cells in passaged FD-AOs, BLM treatment increased the expression of AT1 markers in SPC^+^ cells (Figure 3A). The number of podoplanin-positive (PDPN^+^) cells was evaluated by flow cytometry (FCM) to address the question of whether BLM treatment affected the AT2-AT1 composition ratio in FD-AOs (Figure 3B). Although there was no significant difference in the proportion of PDPN^+^SPC^−^ cells in which AT1-like cells were expected to be included, the number of PDPN^−^SPC^+^ cells decreased while PDPN^+^SPC^+^ cells increased slightly (Figure 3C).

Kobayashi et al. (2020) reported that AT2 cells enter an intermediate, pre-alveolar type-1 transitional cell state (PATS) during the process of differentiating into AT1 cells in mice. Several other reports also supported the presence of AT2-AT1 intermediate cells in pulmonary fibrosis and reported that such intermediate-state cells increased in IPF lungs (Choi et al., 2020; Kobayashi et al., 2020; Strunz et al., 2020). GO enrichment analysis showed that the genes annotated as “Signal transduction by p53 class mediator” and “Epithelial cell differentiation” were enriched in the top 500 DEGs between DMSO-treated and BLM-treated SPC^+^ cells in FD-AOs (Figure 3D). Furthermore, BLM treatment increased the gene expression of the PATS markers in SPC^+^ cells (Figure 3E). Since p53 has been reported as a key transcription factor in the induction of PATS-like cells, we evaluated the co-expression of p53 and SFN, a PATS marker gene that is reported to be upregulated in the IPF lung. IFA showed an increase in the number of p53^+^SFN^+^ epithelial cells in BLM-treated FD-AOs, and the p53^+^SFN^+^ cells appeared to express SPC-GFP weakly or not at all (Figure 3F). These results suggest that PATS-like cells could increase in BLM-treated FD-AOs.

### BLM treatment induces cellular senescence in AT2 cells

**Figure 4:**
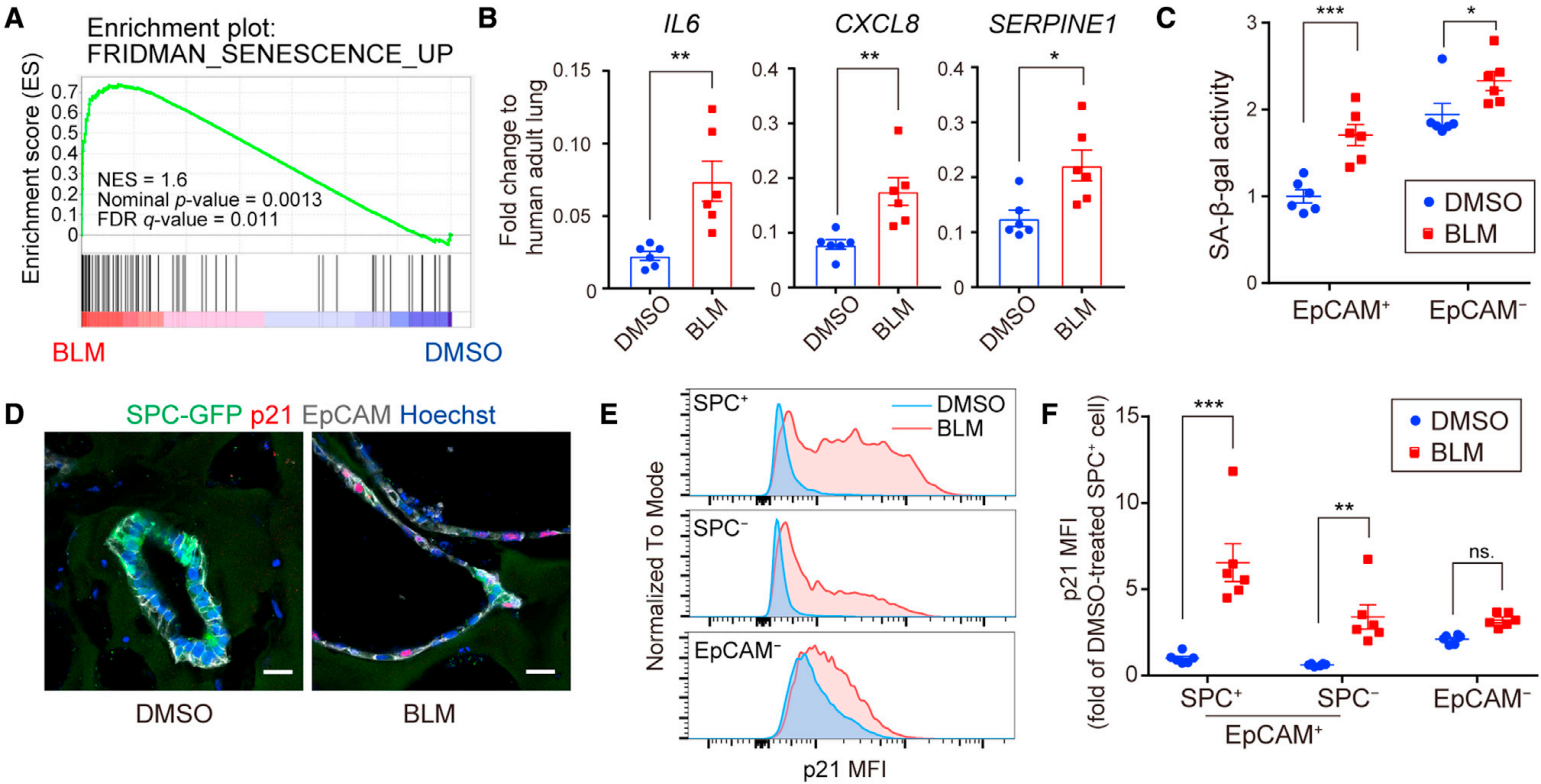
BLM induces cellular senescence in AT2 cells (A) GSEA analysis of “Fridman_senescence_up.” Data from SPC-GFP^+^ cells were used and ranked based on the p value of DESeq2. (B) Expression of the SASP factors in FD-AOs evaluated by qRT-PCR. Gene expression in the normalizer (adult lung RNA control) was set at 1. Data are presented as mean ± SEM (n = 6 independent experiments). Unpaired two-tailed Student’s t test: ^∗^p < 0.05, ^∗∗^p < 0.01. (C) SA-β-gal activity in MACS-separated EpCAM^+^ and EpCAM^−^ cells. Each value was corrected based on protein concentration and expressed as a value relative to DMSO-treated EpCAM^+^ cells. Data are presented as mean ± SEM (n = 6 independent experiments). Two-way ANOVA with Sidak’s multiple comparisons test: ^∗^p < 0.05, ^∗∗∗^p < 0.001; ns, not significant. (D) IFA of FD-AOs. Green, SPC-GFP; red, p21; gray, EpCAM; blue, nuclei (Hoechst). Scale bars, 20 μm. (E and F) p21 expression evaluated by FCM. Data are presented as mean ± SEM (n = 6 independent experiments). Two-way ANOVA with Sidak’s multiple comparisons test: ^∗∗^p < 0.01, ^∗^∗∗p < 0.001; ns, not significant.

GO enrichment analysis of the DEGs in BLM-treated SPC^+^ cells showed that genes associated with inflammation were enriched and those associated with the GO term “Aging” were detected (Figure 3D). In addition, “Aging” was also listed when we performed GO enrichment analysis using IPF AT2 cell-related genes recapitulated in BLM-treated SPC^+^ cells (Figures S4A and S4B) (Reyfman et al., 2019; Rouillard et al., 2016). It has been reported that cellular senescence of alveolar epithelial cells occurs in the IPF lung and that it could be a trigger for pulmonary fibrosis (Reyfman et al., 2019; Yao et al., 2021). Therefore, we evaluated whether the present BLM-induced model could be used as a senescence model of AT2 cells. GSEA showed that “cellular senescence” was significantly upregulated (nominal p value = 0.0013, FDR q value = 0.011) (Figure 4A). It has been reported that senescent cells secrete various pro-inflammatory cytokines, growth factors, and proteases that enhance cellular senescence in an autocrine and paracrine manner. These features of senescent cells are called senescence-associated secretory phenotype (SASP) (Coppé et al., 2010). Expression of major SASP factors *IL6*, *CXCL8*, and *SERPINE1* was increased in BLM-treated FD-AOs (Figure 4B). To evaluate the extent of senescence in each population, we isolated epithelial cells and fibroblasts from FD-AOs and measured the activity of senescence-associated β-galactosidase (SA-β-gal). SA-β-gal activity increased in both populations upon BLM treatment, but the increase was greater in epithelial cells than in fibroblasts (Figure 4C). Consistent with the SA-β-gal activity, IFA showed an increase in the number of p21^+^ epithelial cells (Figure 4D). Quantitative analysis using FCM showed that p21 was upregulated upon BLM treatment in epithelial cells, especially in AT2 cells (Figures 4E and 4F). Furthermore, BLM treatment also induced cellular senescence in FF-AOs (Figure S3I). These results suggest that BLM directly induces cellular senescence in alveolar epithelial cells and that AT2 cells could be more vulnerable to DNA damage.

### Inhibition of ALK5 ameliorates BLM-induced abnormality in epithelial cells in FD-AOs

**Figure 5:**
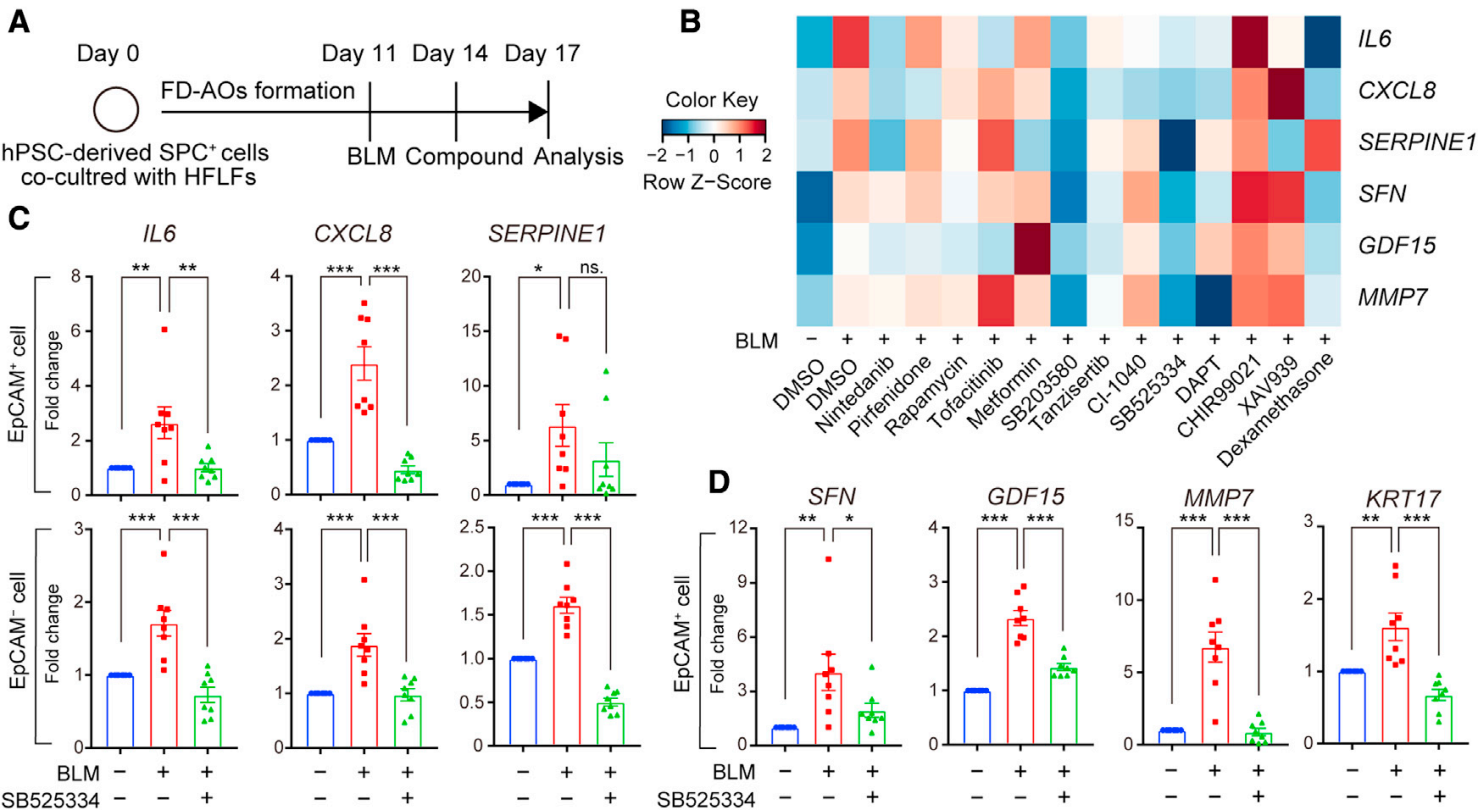
Compound screening identifies SB525334, an ALK5 inhibitor, which ameliorates BLM-induced abnormality of epithelial cells in FD-AOs (A) Schematic overview of compound treatment. (B) Heatmap indicating *Z* scores of the SASP factors (*IL6*, *CXCL8*, *SERPINE1*) and PATS markers (*SFN*, *GDF15*, *MMP7*). Raw data were measured by qRT-PCR in whole organoids. The *Z* scores were calculated using the averaged value of fold change relative to the BLM-treated sample in each experiment (n ≥ 3 independent experiments). (C) Expression of the SASP factors in EpCAM^+^ and EpCAM^−^ cells separated from FD-AOs measured by qRT-PCR and normalized to each control. (D) Expression of the PATS markers in EpCAM^+^ cells separated from FD-AOs. Data are presented as mean ± SEM (n = 8 independent experiments). One-way ANOVA with Dunnett’s multiple comparisons test: ^∗^p < 0.05, ^∗∗^p < 0.01, ^∗^∗∗p < 0.001; ns, not significant.

In a search for key signaling pathways that could be therapeutically used to ameliorate SASP and PATS in BLM-treated FD-AOs, compounds that have been reported to affect cellular senescence, inflammation, or the program of AT2-to-AT1 cell differentiation were selected for screening (Table S3) (Das et al., 2007; Kanagaki et al., 2021a; Paez-Ribes et al., 2019; Yamamoto et al., 2017). A p38 inhibitor, SB203580, and an ALK5 inhibitor, SB525334, were found to suppress the markers of SASP (*IL6*, *CXCL8*, *SERPINE1*) and PATS (*SFN*, *GDF15*, *MMP7*) in BLM-treated FD-AOs (Figures 5A and 5B). Since ATP-competitive kinase inhibitors have been reported to exhibit a number of off-target effects (Knight and Shokat, 2005), a validation study was conducted using compounds with the same target but different structures (Figures S5A and S5B). All ALK5 inhibitors, but not all p38 inhibitors, suppressed the markers of SASP and PATS in BLM-treated FD-AOs (Figure S5C). Therefore, we focused on ALK5 inhibitors for further studies. Since these studies assessed the whole organoids, we separated epithelial cells and fibroblasts to investigate the efficacy of the ALK5 inhibitors in each population. SB525334 suppressed the expression of SASP markers in both populations (Figure 5C). The inhibitory effect was also observed for the PATS marker expression in epithelial cells of FD-AOs (Figure 5D) and in the MMP7 concentration in the culture supernatant (Table S4).

### Inhibition of ALK5 promotes AT1 cell differentiation in BLM-treated FD-AOs

**Figure 6:**
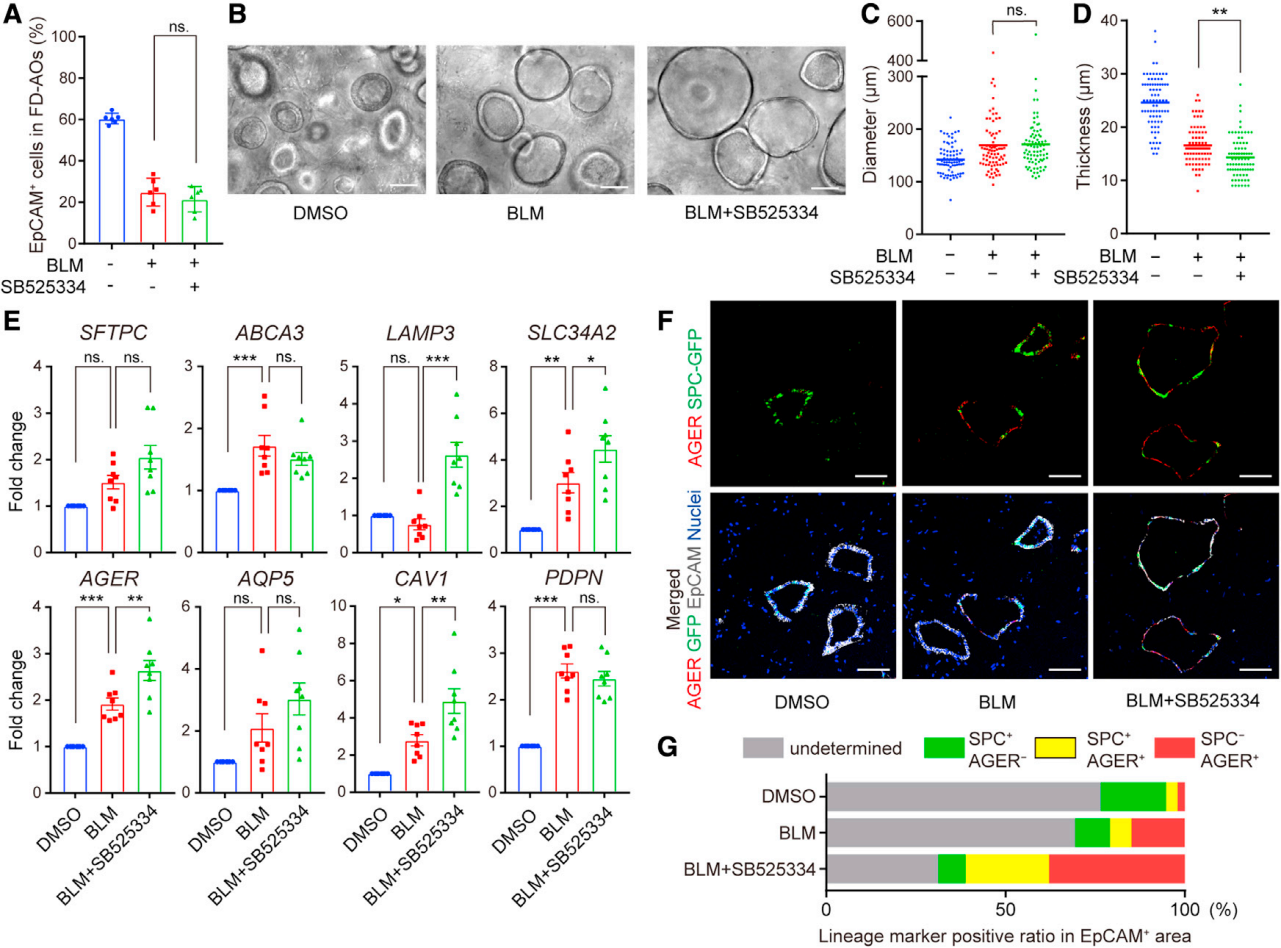
Inhibition of ALK5 promotes AT1 cell differentiation in BLM-treated FD-AOs (A) Analyses of cell component ratio in FD-AOs using FCM. Data are presented as mean ± SEM (n = 6 independent experiments). (B) Live-cell imaging of epithelial cells in FD-AOs. Scale bars, 100 μm. (C and D) Quantification of diameter and thickness of alveolar spheroids. Data are presented as mean ± SEM (n = 80 spheroids from 4 independent experiments). (E) Expression of AT2 cell markers (*SFTPC*, *ABCA3*, *SLC34A2*, *LAMP3*) and AT1 cell markers (*AQP5*, *AGER*, *CAV1*, *PDPN*) in EpCAM^+^ cells measured by qRT-PCR. Data are presented as mean ± SEM (n = 8 independent experiments). (F) IFA of FD-AOs. Green, SPC-GFP; red, AGER; gray, EpCAM; blue, nuclei (Hoechst). Scale bars, 100 μm. (G) Ratio of lineage marker-positive area to EpCAM^+^ area. Data are presented as mean (n = 3 independent experiments). One-way ANOVA with Dunnett’s multiple comparisons test: ^∗^p < 0.05, ^∗∗^p < 0.01, ^∗^∗∗p < 0.001; ns, not significant.

We evaluated the effect of ALK5 inhibition on differentiation states of alveolar epithelial cells in BLM-treated FD-AOs. The proportion of EpCAM^+^ cells and the diameter of alveolar spheroids in BLM-treated FD-AOs were not changed by ALK5 inhibition (Figures 6A–6C), but the thickness of alveolar spheroids was decreased (Figure 6D). ALK5 inhibition upregulated AT1 cell marker expression other than that for *PDPN* in BLM-treated FD-AOs while it did not seem to downregulate AT2 cell marker expression in EpCAM^+^ cells (Figure 6E). Indeed, IFA showed that the AGER^+^ area of the EpCAM^+^ area was increased by BLM treatment and further enhanced by ALK5 inhibition (Figures 6F and 6G). These results suggest that ALK5 inhibitors ameliorate the expression of both SASP and PATS markers while maintaining or protecting the differentiation states of alveolar epithelial cells.

### Fibrogenic changes in BLM-treated FD-AOs are mediated by TGFβ1 signaling from epithelial cells

**Figure 7:**
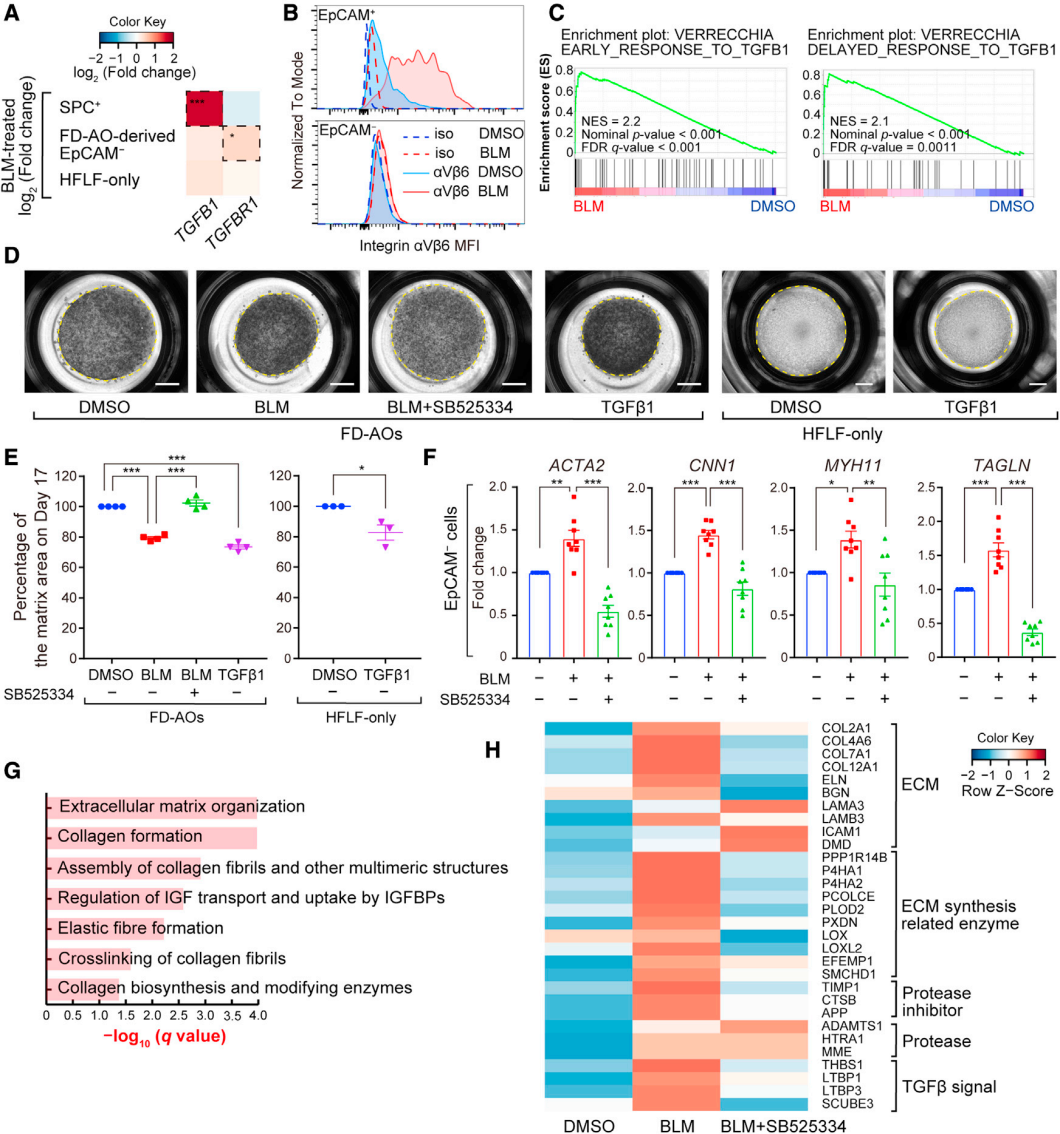
Fibrogenic changes are mediated by alveolar epithelial cell-derived TGFβ1 signaling in BLM-treated FD-AOs (A) Heatmap indicating log_2_(fold change) of *TGFB1* and *TGFBR1* in each population calculated from RNA-seq counts using DESeq2. DESeq2 adjusted: ^∗^p < 0.05, ^∗∗∗^p < 0.001. (B) Cell-surface levels of integrin αVβ6 in EpCAM^+^ and EpCAM^−^ cell populations were measured by FCM. (C) GSEA analysis of “Verrecchia_early/delayed response to TGFB1.” Data from EpCAM^−^ cells separated from FD-AOs were used and ranked based on the p value of DESeq2. (D) Whole-well imaging of cultivation matrices at day 17. Each well was treated with BLM from day 11 to day 14 and with 1 μM SB525334 or 3 ng/mL active TGFβ1 from day 14 to day 17. Scale bars, 2 mm. (E) Quantification of matrix area. Data are presented as mean ± SEM (n = 3 or 4 independent experiments). (F) Expression of contractile genes in fibroblasts separated from FD-AOs measured by qRT-PCR and normalized to each control. Data are presented as mean ± SEM (n = 8 independent experiments). (G) Pathway enrichment analysis using the Reactome software of 427 proteins upregulated by BLM. Proteomic analysis was performed on whole cultivation matrices including cells, and the threshold for upregulation was set to log_2_(fold change) > 0.4. (H) Efficacy of SB525334 on all 30 proteins annotated with “Extracellular matrix organization” that were upregulated by BLM. Heatmap indicating *Z* score of each protein. The log_2_(fold change) was used to calculate the *Z* scores. One-way ANOVA with Tukey’s multiple comparisons test: ^∗^p < 0.05, ^∗∗^p < 0.01, ^∗∗∗^p < 0.001; ns, not significant.

Since ALK5 inhibitors suppress transforming growth factor β (TGFβ) signaling, we asked whether TGFβ signaling was activated in BLM-treated FD-AOs. We reanalyzed the RNA-seq data and observed that *TGFB1* expression was significantly upregulated in SPC^+^ cells upon BLM treatment, while there was no significant change in the expression of *TGFB1* in fibroblasts derived from FD-AOs or HFLF-only culture (Figure 7A). In contrast, *TGFBR1* was significantly upregulated only in fibroblasts isolated from BLM-treated FD-AOs.

TGFβ1 is produced and secreted in an inactive latent form that cannot bind to its receptors and must be activated by certain factors (Munger and Sheppard, 2011). Integrin αVβ6 is a TGFβ-activating integrin whose expression is upregulated in injured epithelial cells, and BLM-induced fibrosis is suppressed in integrin αVβ6-deficient mice (Munger et al., 1999). Integrin αVβ6 has been reported to be upregulated in IPF lungs (Horan et al., 2008), and the IPF cell atlas (Neumark et al., 2020) shows that integrin αVβ6 has particularly high expression in the abnormal epithelial cells called “aberrant basaloid cells” found in IPF lungs (Figure S6A). In our model, integrin αVβ6 was upregulated in epithelial cells upon BLM treatment (Figure 7B) and highly expressed in SPC^+^PDPN^+^ cells (Figure S6B). The integrin αVβ6 protein is composed of αV and β6 subunits. Its expression is regulated by the *ITGB6* gene, and was shown to be suppressed by SB525334 in this model (Figures S6C and S6D). Furthermore, GSK3008348, an integrin αVβ6 antagonist, partially suppressed the expression of SASP and PATS marker genes in BLM-treated FD-AOs (Figures S6E and S6F). BLM treatment also upregulated the mRNA expression of *TGFB1* and *ITGB6* in FF-AOs (Figure S7). Although active TGFβ1 could not be detected in the culture supernatant of BLM-treated FD-AOs (Table S4), these results suggest that alveolar epithelial cells could be a source of TGFβ and play a role as local activator of TGFβ (Wu et al., 2020).

GSEA analysis showed that fibroblasts received the TGFβ1 signal in BLM-treated FD-AOs (Figure 7C). Functionally, BLM-induced contraction of FD-AOs was ameliorated by SB525334 and GSK3008348 (Figures S6G and S6H). Furthermore, addition of active TGFβ1 recapitulated the global contraction of the matrices in FD-AOs or HFLF-only culture (Figures 7D and 7E). In transcriptome analysis, contractile genes *ACTA2*, *CNN1*, *MYH11*, and *TAGLN* (Popova et al., 2010) were downregulated by SB525334 in fibroblasts separated from BLM-treated FD-AOs (Figure 7F). Finally, proteomic analysis of FD-AOs, including the cultivation matrix, was performed to evaluate the accumulation of extracellular matrix, which is strong evidence of fibrosis (Table S5). Pathway enrichment analysis using Reactome software (Jassal et al., 2020) showed that proteins annotated to “Extracellular matrix organization” and “Collagen formation” were enriched in proteins upregulated upon BLM treatment (Figure 7G). We then evaluated the efficacy of SB525334 on all 30 proteins annotated to “Extracellular matrix organization” that were upregulated upon BLM treatment. These results showed that SB525334 did not downregulate the expression of adhesion factors and proteases but did downregulate the expression of collagen and its synthases as well as proteins related to TGFβ signaling (Figure 7H).

## Discussion

Epithelial-mesenchymal interactions have long been reported to play important roles in various disorders that depend on fibrogenesis, including IPF (Katzen and Beers, 2020), and in site-specific tissue regeneration (Yamaguchi et al., 2005). However, there are few practical human *in vitro* models of pulmonary fibrosis because of the difficulty of culturing alveolar epithelial cells. Therefore, little progress has been made toward the study of human pulmonary fibrosis or the identification of therapeutic agents for it that target epithelial cells (Spagnolo et al., 2020). Recently, it has been reported that AT2 cells isolated from human tissues could be cultured over a long term while maintaining their functions (Katsura et al., 2020; Youk et al., 2020). These cells are expected to be a source for complex lung disease models such as IPF that presumably require co-culture experiments. However, primary cells could have a large lot-to-lot variability, which poses a challenge in utilizing them in continuous screening over a long period of time. Furthermore, because human primary cells have been difficult to modify genetically via cloning, hPSC-derived cells have been advantageous in analyzing detailed molecular mechanisms of differentiation and disease models.

In this study, we demonstrated that hPSC-derived FD-AOs could be beneficial for screening various chemicals to find potential therapeutic targets for pulmonary fibrosis, targeting alveolar epithelial cells in particular. BLM, a DNA-damaging agent, induced cellular senescence in epithelial cells, especially in AT2 cells in FD-AOs, which is consistent with the presence of cellular senescence in AT2 cells in the IPF lung (Reyfman et al., 2019). In addition, the homeostasis of lipid metabolism is reported to be altered in aged lungs (Angelidis et al., 2019). We speculate that phospholipid accumulation of AT2 cells could be an indicator of aging or senescence, although further validation studies are needed. Several groups have reported the presence of transitionally differentiated alveolar epithelial cells as an intermediate cellular state that is associated with the conversion from AT2 cells to AT1 cells in regenerating lungs (Choi et al., 2020; Kobayashi et al., 2020; Strunz et al., 2020). An increased number of these AT2-AT1 intermediate-like cells, with p53-mediated cellular senescence as a common phenotype, could be present in the IPF lung. We showed that our BLM-treated FD-AOs could be a novel tool to study the abnormally differentiated state of these alveolar epithelial cells in IPF. While screening for signaling inhibitors, we found that ALK5 inhibitors ameliorated phenotypes of fibrogenesis, such as inflammatory responses and AT2-AT1 intermediate-like states that are associated with epithelial senescence, inducing the expression of AT1 cell markers in FD-AOs. This is consistent with the recent finding that inhibition of TGFβ during late stages of the differentiation of AT2 to AT1 cells leads to an increase in AT1 cell markers in a two-dimensional culture model of rat AT2 cells (Riemondy et al., 2019). Furthermore, the effects of TGFβ in the present study may be explained by the presence of a positive feedback loop in TGFβ-secreting senescent AT2 cells, which reinforces cellular senescence in an autocrine and paracrine manner (Hubackova et al., 2012). The secreted TGFβ is activated by integrin αVβ6 of alveolar epithelial cells, especially in cells present in the AT2-AT1 intermediate states, which in turn strengthens the signaling to fibroblasts.

There are three limitations to our model to study epithelial-mesenchymal interactions related to the pathogenesis of IPF. The first is that the chemically induced model can act on both epithelial cells and fibroblasts in FD-AOs. Although BLM has a stronger effect on epithelial cells than on fibroblasts in FD-AOs, it is unclear whether alveolar epithelial cell-specific damage could dominantly activate fibroblasts in FD-AOs. Genetically modified or patient-specific hPSC models that can produce damage specifically to epithelial cells are required for future studies. Second, our FD-AOs contain undetermined epithelial lineage cells (Kanagaki et al., 2021a), and the alveolar epithelial cells are relatively immature compared with adult alveolar epithelial cells. Those undetermined epithelial cells need to be analyzed in detail or be reduced by modification of culture conditions in future studies. The increase in PDPN^+^SPC^+^ cells in BLM-treated FD-AOs might reflect the immaturity of human PSC-derived AT2 cells in the present study, as we previously reported that the transcriptome of FD-AO-derived AT2 cells is more similar to that of a majority of *Sftpc*^+^ cells isolated from embryonic-day-18.5 mice than to that of cells from adult mice (Yamamoto et al., 2017). Therefore, further evaluation is needed to determine whether the increase in PDPN^+^SPC^+^ cells in BLM-treated FD-AOs is indicative of the increase of transdifferentiating cells upon injury (Paris et al., 2020) or of bipotent cells present during development (Desai et al., 2014). Third, the pathogenesis of BLM-treated FD-AOs was relatively weak compared with that of cells in *in vivo* mouse models. We could not fully recapitulate PATS marker gene expression (such as *SOX4* and *CLDN4*) in BLM-treated FD-AOs. Excessive mechanical stress (Wu et al., 2020) and sustained inflammatory response to inflammatory cytokines from immune cells (Choi et al., 2020) have been reported to impair the differentiation program of AT2 cells, which could contribute to the pathogenesis of pulmonary fibrosis. Therefore, a device that mimics lung stretching or interaction with immune cells may adequately address this problem.

In conclusion, we developed an *in vitro* model of pulmonary fibrosis using hPSC-derived alveolar organoids that can be beneficial for screening potential therapeutic agents for IPF. In this model, we observed epithelial cell-dependent BLM-induced cellular senescence in AT2 cells and abnormally differentiated intermediate-state AT2-AT1 cells, along with fibroblast activation, organoid contraction, and extracellular matrix accumulation, similar to the fibrogenesis of IPF. We believe that an *in vitro* recapitulation of pulmonary fibrosis may accelerate not only the understanding the IPF pathogenesis but also drug discovery to help in the treatment of the as yet incurable IPF.

## Experimental procedures

### Culture of hPSCs and human lung fibroblasts

All cells in the present study were cultured under 5% CO_2_ at 37°C. The hPSCs were cultured as previously described (Yamamoto et al., 2017). In brief, SPC-GFP reporter hPSCs (Gotoh et al., 2014), 585A1 (RIKEN BRC #HPS0354), and 648A1 (RIKEN BRC #HPS0360) were maintained in Essential 8 medium (Thermo Fisher Scientific) or mTeSR Plus (STEMCELL Technologies) (Okita et al., 2013). HFLFs (17.5 weeks of gestation; DV Biologics #PP002-F-1349), MRC-5 (14 weeks of gestation; ATCC #CCL-171), and TIG-1-20 (20 weeks of gestation; JCRB Cell Bank #JCRB0501) were cultured in Dulbecco’s modified Eagle’s medium (Nacalai Tesque) supplemented with 10% fetal bovine serum (FBS; Sigma-Aldrich #F7524) and 50 U/mL penicillin-streptomycin (P-S; Thermo Fisher Scientific). LL47 (16 years of age; ATCC #CCL-135) were cultured in Ham's F-12K (Fujifilm Wako) supplemented with 15% FBS and 50 U/mL P-S. SPC-GFP reporter hPSCs and HFLFs were used for all experiments except those described in Figure S1. The use of 585A1 and 648A1 hPSCs was approved by the Ethics Committee of Kyoto University Graduate School and Faculty of Medicine. The use of other cells was exempted from ethical approval.

### Maintenance of hPSC-derived AT2 cells in FD-AOs

hPSC-derived SPC-GFP^+^ cells were isolated from FD-AOs using fluorescence-activated cell sorting (FACS) with APC-conjugated mouse anti-human EpCAM antibody (1:100, Miltenyi Biotec #130-113-260) as described previously (Yamamoto et al., 2017). FACS-sorted SPC-GFP^+^ cells (1 × 10^4^) were mixed with 5 × 10^5^ precultured lung fibroblasts in 200 μL of 50% growth factor reduced Matrigel (Corning) diluted with DCIK medium (Yamamoto et al., 2017) supplemented with 10 μM Y-27632 (LC Laboratories). Approximately 200 μL of the mixed cells was placed on a 12-well cell culture insert (Corning), and 1 mL of DCIK medium containing 10 μM Y-27632 was added to the lower chamber. In the 24-well format, half the fluid volume was used. FD-AOs were cultured for 14 days, and the medium in the lower chamber was replaced with DCIK medium every 2–3 days. SPC-GFP^+^ cells were isolated every 14 days and repeatedly cultured in FD-AOs to maintain the SPC-GFP^+^ cells. The FD-AOs were used with FACS-sorted SPC-GFP^+^ cells passaged 1–8 times. In this study, a BD FACSAria III Cell Sorter (Becton Dickinson) was used for FCM and FACS.

### BLM and compound treatment in FD-AOs

SPC-GFP^+^ cell-derived FD-AOs were treated with 3 μg/mL BLM (Nippon Kayaku) in the lower chamber medium from day 11 to day 14. BLM was washed out on day 14 with PBS (Nacalai Tesque), and the FD-AOs were cultured in dexamethasone-free DCIK medium from day 14 to day 17. Each compound used for screening (Table S3) or with 3 ng/mL active TGFβ1 (Bio-Techne, #7754-BH) was supplemented in the medium from day 14 to day 17.

### Statistical analysis

Data are presented as mean ± standard error of the mean (SEM). The number of biological replicates and statistical tests are described in each figure legend. All statistical tests were performed using Prism7 software (GraphPad). p values of < 0.05 were considered statistically significant.

### Data and code availability

The accession numbers for the sequencing raw data reported in the present study are GEO: GSE172121 and GSE172122.

## Author contributions

Conceptualization, T.S., S.K., R.M., K.T., and S.G.; methodology, T.S. and S.K.; software, T.S. and K. Moriguchi; validation, A.M.; formal analysis, T.S. and S.G.; investigation, T.S., K. Moriguchi, A.M., and S.G.; resources, K.N., M.T., and S.G.; writing, T.S. and S.G.; supervision, T.H., K. Murakami, and M.H.

## Conflicts of interests

T.S., S.K., K. Moriguchi, A.M., K.N., and K. Murakami are employees, received research funding, and are shareholders of Kyorin Pharmaceutical Co., Ltd. M.H. and S.G. are founders and shareholders of HiLung Inc. M.H. received research funding from Kyorin Pharmaceutical. S.G. is listed as one of the inventors of Kyoto University’s patents related to the method of generating alveolar organoids. The other authors declare no competing interests.

## Supplemental information

Document S1. Supplemental experimental procedures, Figures S1–S7, and Tables S3, S4, S6, S7

Table S1. RNA-seq processed data of fibroblasts under DMSO and BLM treatment with or without epithelial cells, related to Figure 1

Table S2. RNA-seq processed data of SPC^+^ cells under DMSO and BLM treatment, related to Figures 3 and 4

Table S5. Proteomic data of whole cultivation matrices including cells under DMSO, BLM, and BLM with SB525334 treatment, related to Figure 7

Document S2. Article plus supplemental information
