# Supplementary material for: Disease modeling of pulmonary fibrosis using human pluripotent stem cell-derived alveolar organoids
Source: Stem Cell Reports. 2021 Nov 18;16(12):2973–87. doi: 10.1016/j.stemcr.2021.10.015 (PMC8693665; doi:10.1016/j.stemcr.2021.10.015)
Supplement: Document S1. Supplemental experimental procedures, Figures S1–S7, and Tables S3, S4, S6, S7 [file mmc1.pdf]

**Stem Cell Reports, Volume *16***

## Supplemental Information

# Disease modeling of pulmonary fibrosis using human pluripotent stem cell-derived alveolar organoids

**Takahiro Suezawa, Shuhei Kanagaki, Keita Moriguchi, Atsushi Masui, Kazuhisa Nakao, Masayasu Toyomoto, Koji Tamai, Ryuta Mikawa, Toyohiro Hirai, Koji Murakami, Masatoshi Hagiwara, and Shimpei Gotoh**

## SUPPLEMENTAL INFORMATION

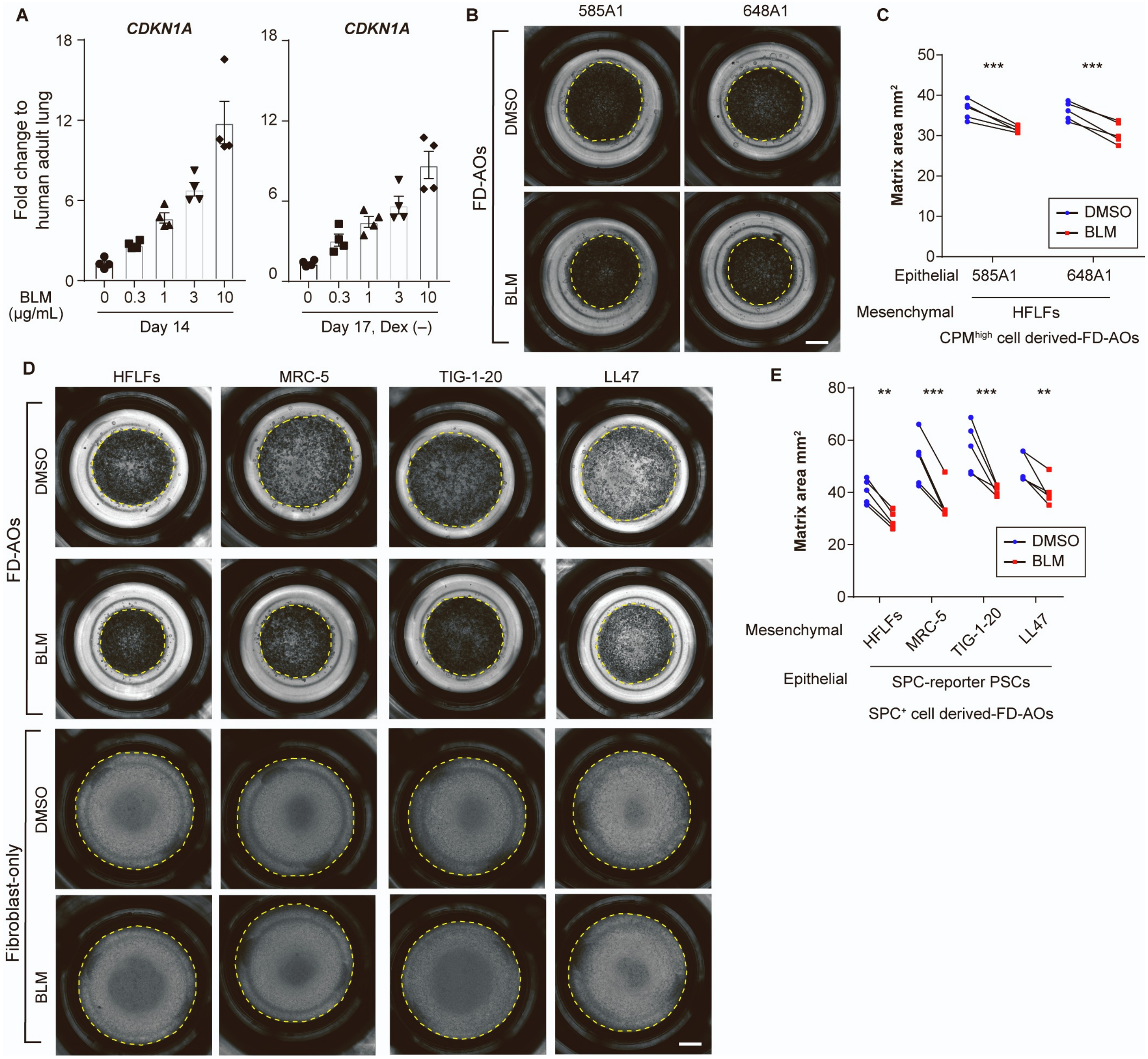


**Figure S1. Optimization of BLM concentration and assay validation using other cell lines. Related to Figure 1.**

(A) Bleomycin (BLM) dose dependency in fibroblast-dependent alveolar organoids (FD-AOs) on Day 14 and 17. Expression levels of *CDKN1A* were evaluated by qRT-PCR. Gene expression in the normalizer (adult lung RNA control) was set at 1. Data are presented as mean ± SEM ($n$ = 4 independent experiment). Dex: dexamethasone. (B–E) Whole-well imaging and quantification of the area of cultivation matrices of fibroblast or 3D-cultured fibroblasts on Day 17. Scale bars, 2 mm. Data are mean ± SEM ($n$ = 5 independent experiments). Two-way repeated measures ANOVA with Sidak's multiple comparisons test: **$P$ < 0.01, ***$P$ < 0.001.

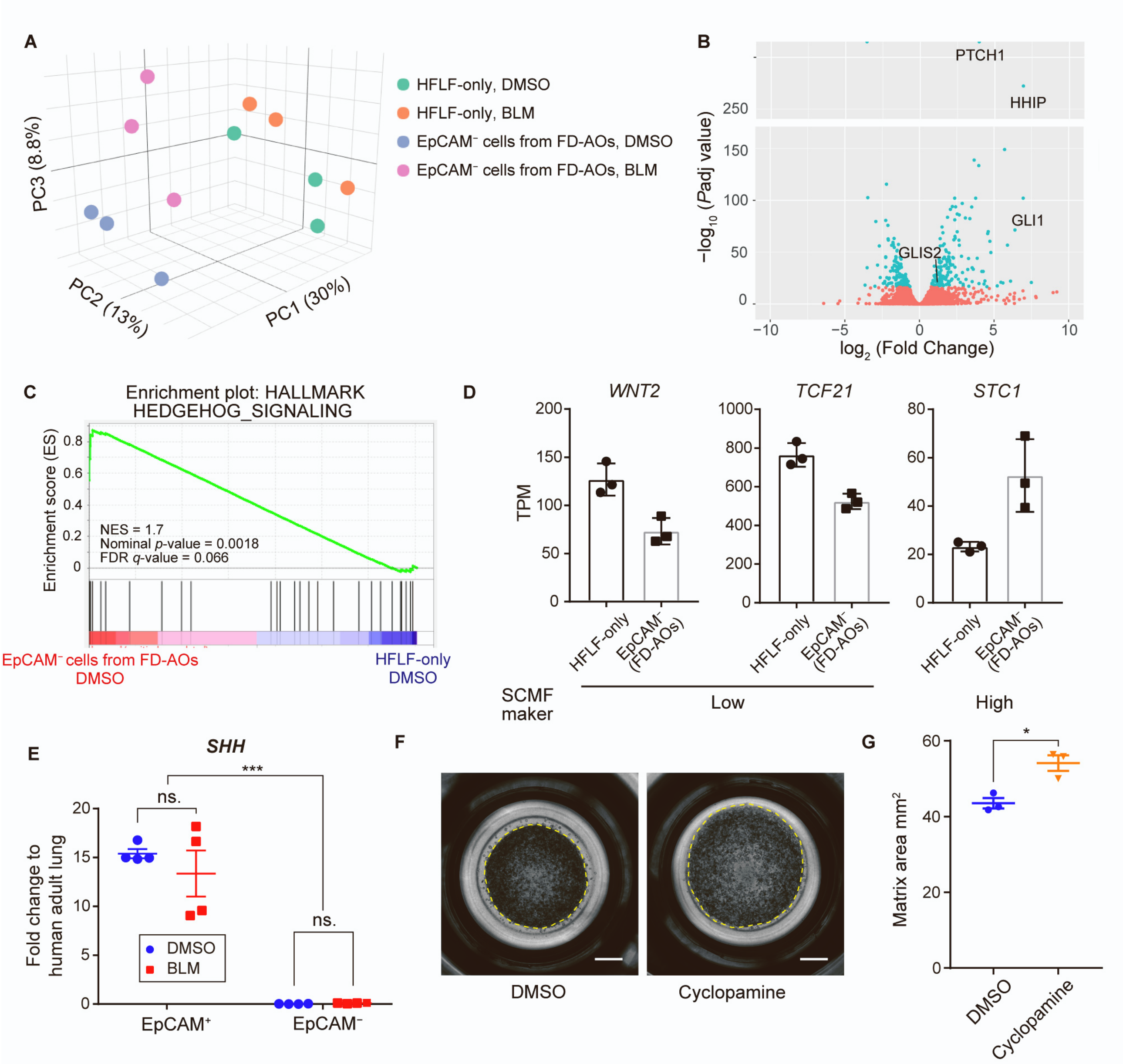


**Figure S2. Hedgehog signal from alveolar epithelial cells contributes to matrix shrinkage of FD-AOs in DMSO-treated conditions. Related to Figure1.**

(A) Principal component analysis (PCA) of the transcriptome of fibroblasts under DMSO and BLM treatment with or without epithelial cells. The $\log_2$ (TPM values) were used for PCA. (B) Volcano plot obtained from the DESeq2 analysis between fibroblasts from FD-AOs and those from HFLF-only culture. (C) GSEA pre-ranked test to Hallmark_Hedgehog_signaling. Data from fibroblasts with or without epithelial cells were used and ranked based on the *p*-values calculated by DESeq2. (D) The gene expression of secondary crest myofibroblast (SCMF) markers (Zepp et al., 2021). The SCMF low-expression markers are *WNT2* and *TCF21*; the SCMF high-expression marker is *STC1*. The RNA-seq data with or without epithelial cells were used. (E)

Expression levels of *SHH* in EpCMA$^+$ and EpCAM$^-$ cells separated from FD-AOs measured by qRT-PCR. Gene expression in the normalizer (adult lung RNA control) was set at 1. Data are presented as mean ± SEM (*n* = 4 independent experiments). Two-way ANOVA with Sidak's multiple comparisons test: ****P* < 0.001. ns.: not significant. (F and G) Whole-well imaging and quantification of the area of cultivation matrices of FD-AOs on Day 14. FD-AOs were treated with 20 μM cyclopamine from Day 11 to 14. Scale bars, 2 mm. Data are presented as mean ± SEM (*n* = 3 independent experiments). Unpaired two-tailed Student's *t* test: **P* < 0.05.

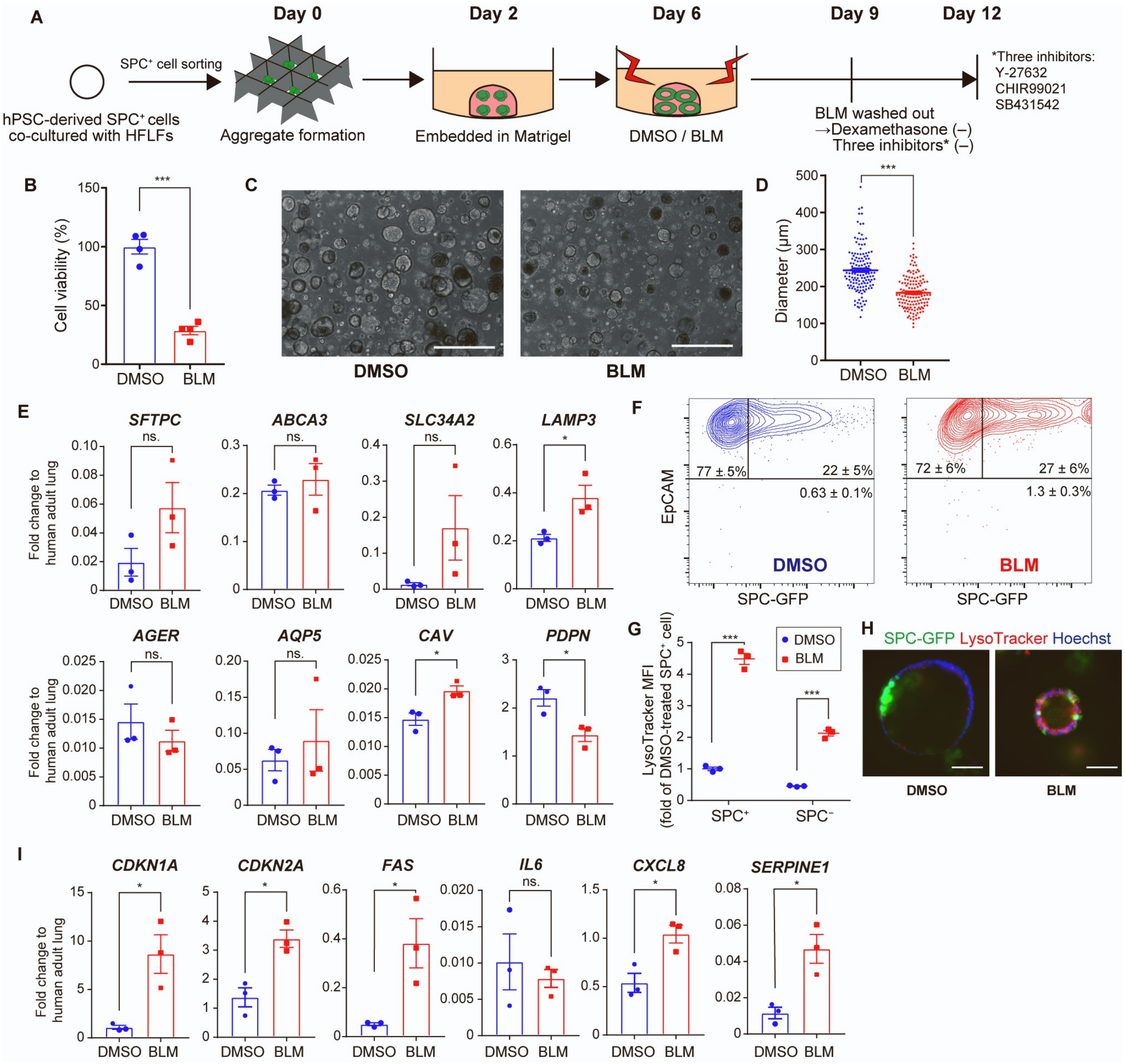


**Figure S3. Response of FF-AOs to BLM treatment. Related to Figures 2 and 4.**

(A) Experimental scheme of BLM treatment of fibroblast-free alveolar organoids (FF-AOs). (B) CellTiter-Glo 3D Cell Viability assay. Data are presented as mean ± SEM ($n = 4$ independent experiments). Unpaired two-tailed Student's $t$ test: ***$P < 0.001$. (C and D) Quantification of alveolar spheroid diameter. Scale bars, 1000 µm. Data are presented as mean ± SEM ($n = 140$ spheres from 7 independent experiments). Unpaired two-tailed Student's $t$ test: ***$P < 0.001$. (E) Expression levels of AT2 (*SFTPC*, *ABCA3, SLC34A2* and *LAMP3*) and AT1 (*AGER*, *AQP5*, *CAV1* and *PDPN*) cell markers in FF-AOs evaluated by qRT-PCR. Gene expression in the normalizer (adult lung RNA control) was set at 1. Data are presented as mean ± SEM ($n = 3$).

Unpaired two-tailed Student's $t$ test: *$P < 0.05$. ns.: not significant. (F and G) Flow cytometric analysis of BLM-treated FF-AOs. Mean fluorescence intensity (MFI) of LysoTracker. Data are mean ± SEM ($n = 3$ independent experiments). Two-way ANOVA with Sidak's multiple comparisons test: ***$P < 0.001$. (H) Live cell imaging of FF-AOs. Green, SPC-GFP; red, LysoTracker; blue, nuclei (Hoechst). Scale bars, 100 µm. (I) Expression levels of senescence markers in FF-AOs evaluated by qRT-PCR. Gene expression in the normalizer (adult lung RNA control) was set at 1. Data are presented as mean ± SEM ($n = 3$). Unpaired two-tailed Student's $t$ test: *$P < 0.05$. ns.: not significant.

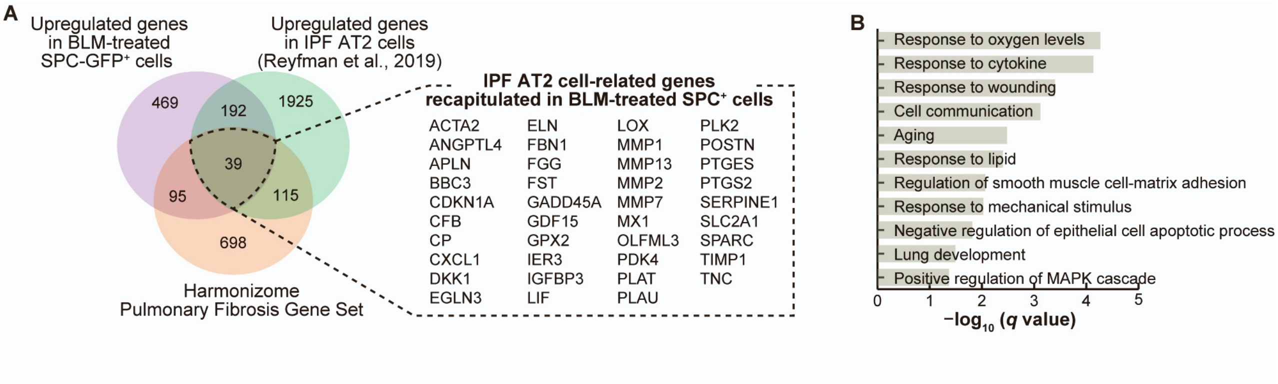


**Figure S4. Transcriptomic comparison of isolated AT2 cells from BLM-treated FD-AOs and those from the IPF lung. Related to Figures 3 and 4.**

(A) Among the 947 genes annotated as "pulmonary fibrosis" in the harmonizome database (Rouillard et al., 2016), we extracted genes that were commonly upregulated in AT2 cells of BLM-treated FD-AOs and the idiopathic pulmonary fibrosis (IPF) lung (Reyfman et al., 2019). The threshold for upregulation was set to $\log_2$ (fold change) > 1 with adjusted $p$-value < 0.05. (B) GO analysis of the 39 extracted genes.

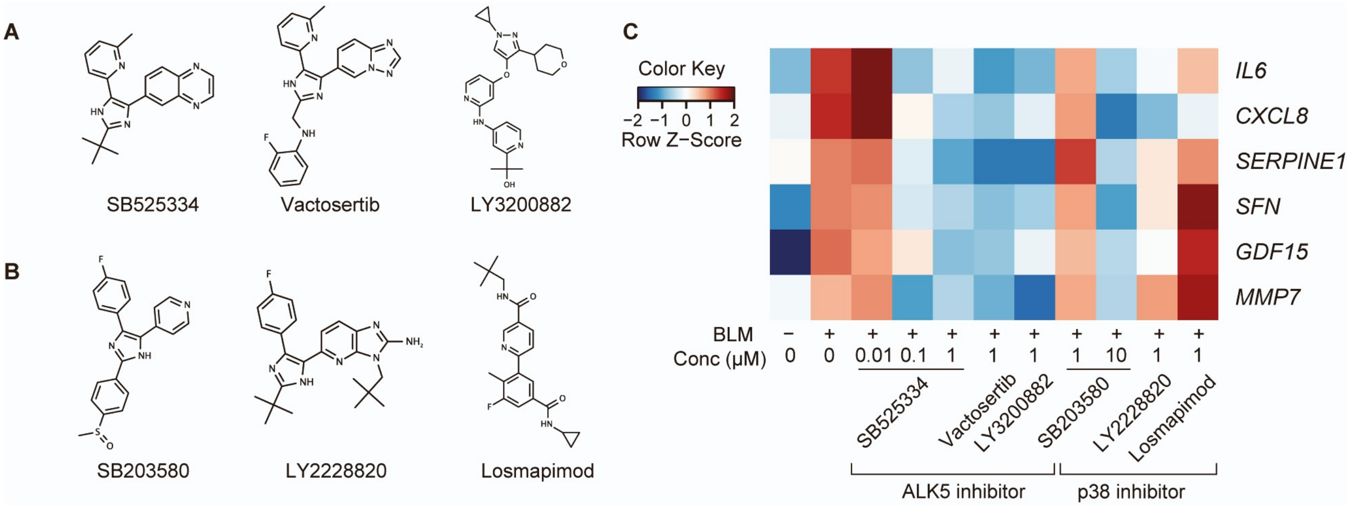


**Figure S5. TGFβ signaling contributes to the pathogenesis of epithelial cells. Related to Figure 5.**

(A) Structures of ALK5 inhibitors. (B) Structures of p38 inhibitors. (C) A validation study of ALK5 and p38 using compounds with the same target but different structures. Heatmap indicating Z-scores of the senescence-associated secretory phenotype (SASP) factors (*IL6*, *CXCL8*, *SERPINE1*) (Coppé et al., 2010) and pre-alveolar type-1 transitional cell state (PATS) markers (*SFN*, *GDF15*, *MMP7*) (Kobayashi et al., 2020). Raw data were measured by qRT-PCR in whole organoids. The Z-scores were calculated using the averaged value of fold change relative to the BLM-treated sample in each experiment ($n$ = 3 independent experiments).

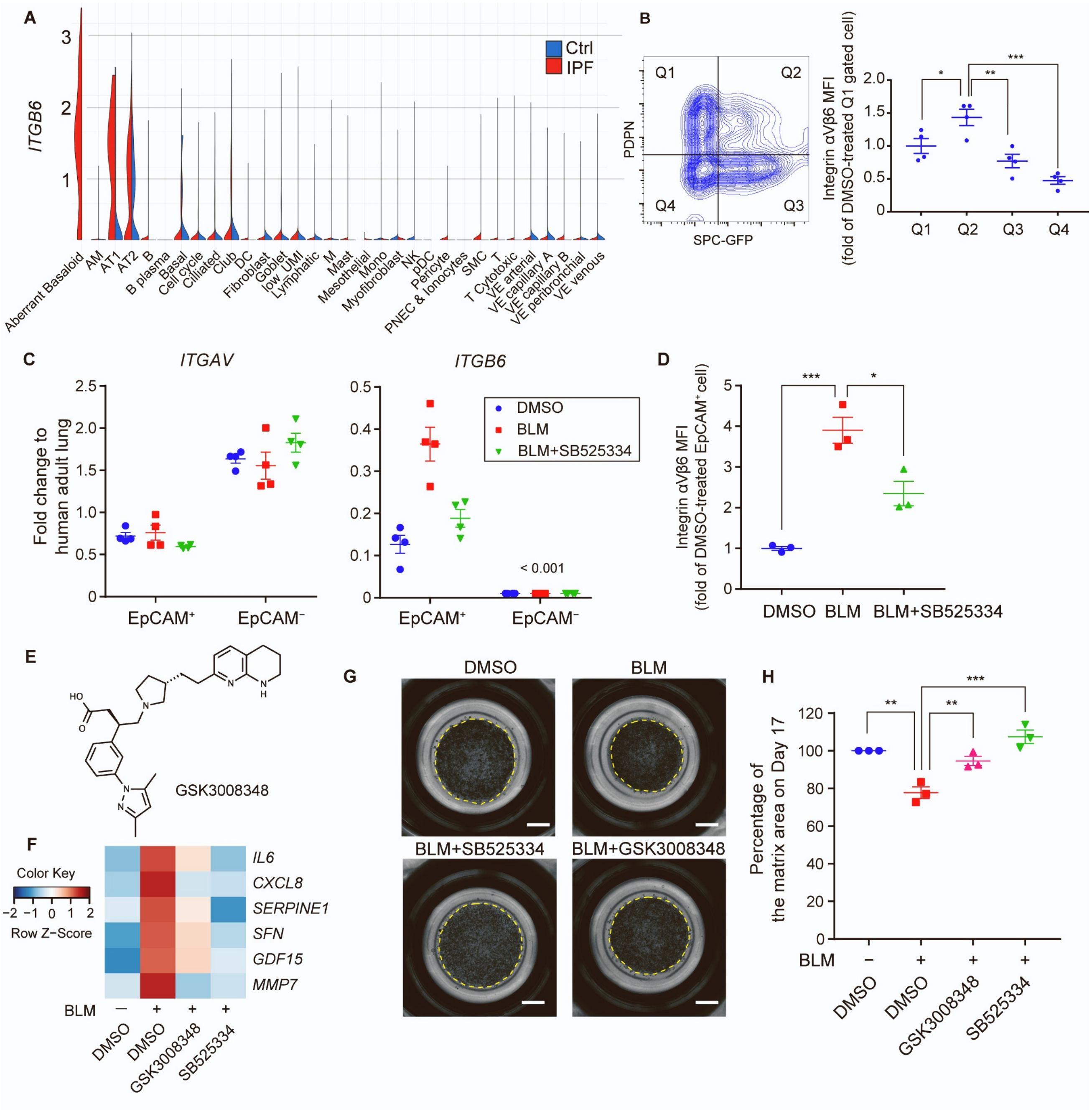


**Figure S6. Integrin αVβ6 is specifically expressed in epithelial cells contributing to BLM-induced fibrogenic changes. Related to Figure 7.**

(A) *ITGB6* expression in IPF using the IPF Cell Atlas (www.ipfcellatlas.com) (Neumark et al., 2020). Data set used was Lafyatis. (B) Flow cytometric analysis of DMSO-treated EpCAM$^+$ cells. Left, gating strategy for isolating each cell population; right, MFI of integrin αVβ6. Data are presented as mean ± SEM ($n$ = 4 independent experiments). (C) Expression levels of *ITGAV* and *ITGB6* in EpCMA$^+$ and EpCAM$^-$ cells separated from FD-AOs measured by qRT-PCR. Gene expression in the normalizer (adult lung RNA control) was set at 1. Data are presented as mean

± SEM ($n$ = 4 independent experiments). (D) MFI of integrin αVβ6 gated on EpCAM+ cells of FD-AOs in each condition. Data are presented as mean ± SEM ($n$ = 3 independent experiments). (E) Structure of GSK3008348, an integrin αVβ6 antagonist. (F) Heatmap indicating Z-scores of the SASP and PATS genes. Raw data were measured by qRT-PCR in whole organoids. The Z-scores were calculated using the averaged value of fold change relative to the BLM-treated sample in each experiment ($n$ = 3 independent experiment). FD-AOs was treated with BLM from Day 11 to 14, and with 1 μM GSK3008348 from Day 14 to 17. (G) Whole-well imaging of cultivation matrices on Day 17. Scale bars, 2 mm. (H) Quantification of matrix area. Data are presented as mean ± SEM ($n$ = 3 independent experiments). One-way ANOVA with Tukey's multiple comparisons test: *$P$ < 0.05, **$P$ < 0.01, ***$P$ < 0.001.

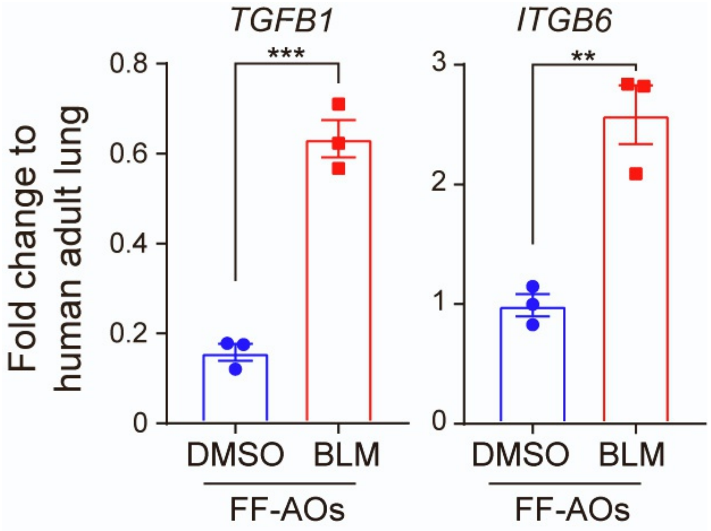


**Figure S7. BLM-treatment activates TGFβ signaling in FF-AOs. Related to Figure 7.**

FF-AOs were treated with BLM form Day 6 to 9 and analyzed on Day 12. *TGFB1* and *ITGB6* in FF-AOs were evaluated by qRT-PCR. Gene expression in the normalizer (adult lung RNA control) was set at 1. Data are presented as mean ± SEM ($n$ = 3). Unpaired two-tailed Student's $t$ test: **$P$ < 0.01, ***$P$ < 0.001.

**Table S1 (Separate file). RNA-seq processed data of fibroblasts under DMSO and BLM treatment with or without epithelial cells. Related to Figure 1.**

**Table S2 (Separate file). RNA-seq processed data of SPC$^+$ cells under DMSO and BLM treatment. Related to Figures 3 and 4.**

**Table S3. Compounds for screening. Related to Figure 4.**

| Compound name | Vendor | Concentration (μM) |
|---|---|---|
| Nintedanib | Cayman CHEMICAL | 1 |
| Pirfenidone | Tokyo Chemical Industry | 500 |
| Rapamycin | Nacalai Tesque | 0.1 |
| Tofacitinib | MedChemExpress | 1 |
| Metformin | Fujifilm Wako | 1000 |
| SB203580 | Adipogen | 1 and 10 |
| Tanzisertib | MedChemExpress | 1 |
| CI-1040 | MedChemExpress | 1 |
| SB525334 | MedChemExpress | 0.01, 0.1 and 1 |
| DAPT | Fujifilm Wako | 10 |
| CHIR99021 | Axon Medchem | 3 |
| XAV939 | Sigma-Aldrich | 10 |
| Dexamethasone | Sigma-Aldrich | 0.05 |
| Vactosertib | MedChemExpress | 1 |
| LY3200882 | MedChemExpress | 1 |
| LY2228820 | ChemScene | 1 |
| Losmapimod | MedChemExpress | 1 |
| GSK3008348 | MedChemExpress | 1 |
| Cyclopamine | Cayman CHEMICAL | 20 |

**Table S4. Secreted molecules in the culture supernatant of FD-AOs on Day 17. Related to Figures 5 and 7.**

Data are presented as mean ± SEM (*n* = 5 independent experiments).

| Stimulus | MMP7 (pg/mL) | Active TGFβ1 (pg/mL) | Total TGFβ1 (pg/mL) |
|---|---|---|---|
| DMSO | 163 ± 19 | nd (< 2 pg/mL) | 220 ± 29 |
| BLM | 262 ± 46 | nd (< 2 pg/mL) | 178 ± 37 |
| BLM + 1 μM SB525334 | 121 ± 20 | nt | nt |

Definition of abbreviations: ‘nd’ not detected; ‘nt’ not tested

**Table S5 (Separate file). Proteomic data of whole cultivation matrices including cells under DMSO, BLM, and BLM with SB525334 treatment. Related to Figure 7.**

**Table S6. Primers for TaqMan qPT-PCR**

| Gene name | Taqman ID |
|---|---|
| *18S rRNA* | Hs99999901_s1 |
| *CDKN1A* | Hs00355782_m1 |
| *SFTPC* | Hs00161628_m1 |
| *IL6* | Hs00985639_m1 |
| *CXCL8* | Hs00174103_m1 |
| *SERPINE1* | Hs00167155_m1 |
| *CDKN2A* | Hs00233365_m1 |
| *FAS* | Hs00236330_m1 |
| *SFN* | Hs00968567_s1 |

| Gene name | Taqman ID |
|---|---|
| *GDF15* | Hs00171132_m1 |
| *MMP7* | Hs01042796_m1 |
| *ACTA2* | Hs00426835_g1 |
| *CNN1* | Hs00959434_m1 |
| *MYH11* | Hs00975796_m1 |
| *TAGLN* | Hs01038777_g1 |
| *ITGAV* | Hs00233808_m1 |
| *ITGB6* | Hs00168458_m1 |
| *TGFB1* | Hs99999918_m1 |

**Table S7. Primer sequences for SYBR green qRT-PCR**

| Gene name | Forward primer sequence | Reverse primer sequence |
|---|---|---|
| *SHH* | CCGAGCGATTTAAGGAACTCACC | AGCGTTCAACTTGTCCTTACACC |
| *ABCA3* | TCTCCTTCAGCTTCATGGTCAG | TGGCTCAGAGTCATCCAGTTG |
| *SLC34A2* | TCGCCACTGTCATCAAGAAG | CTCTGTACGATGAAGGTCATGC |
| *LAMP3* | ACCGATGTCCAACTTCAAGC | TGACACCTTAGGCGGATTTT |
| *AQP5* | CTGTCCATTGGCCTGTCTGTC | GGCTCATACGTGCCTTTGATG |
| *AGER* | GCCACTGGTGCTGAAGTGTA | TGGTCTCCTTTCCATTCCTG |
| *CAV1* | AGGGCAACATCTACAAGCCC | GCCGTCAAAACTGTGTGTCC |
| *PDPN* | TCCAGGAACCAGCGAAGAC | CGTGGACTGTGCTTTCTGA |
| *KRT17* | CTACAGCCAGTACTACAGGACA | AACTTGGTGCGGAAGTCATCA |

## SUPPLEMENTAL EXPERIMENTAL PROCEDURES

### Differentiation of hPSCs into NKX2-1$^+$ lung progenitor cells

The differentiation of hPSCs was performed as previously described (Yamamoto et al., 2017). To differentiate into definitive endoderm (DE), hPSCs were seeded on Geltrex-coated (Thermo Fisher Scientific) plates in RPMI medium (Nacalai Tesque) containing 100 ng/mL activin A (Peprotech), 1 µM CHIR99021 (Axon Medchem), 10 µM Y-27632 (LC Laboratories), 2% B-27 supplement (Thermo Fisher Scientific), and 50 U/mL penicillin–streptomycin (P–S; Thermo Fisher Scientific). Sodium butyrate (Fujifilm Wako) was added to a final concentration of 0.25 mM on the next day. From the second day, the cells were cultured in RPMI medium containing 100 ng/mL activin A, 1 µM CHIR99021, 0.125 mM sodium butyrate, 2% B-27 supplement, and 50 U/mL P–S for 4 days. In the following steps, DMEM/F12 (Thermo Fisher Scientific) containing GlutaMAX (Thermo Fisher Scientific), 2% B-27 supplement, 50 U/ml P–S, 0.05 mg/mL L-ascorbic acid (Fujifilm Wako), and 0.4 mM monothioglycerol (Fujifilm Wako) was used as the basal medium. The DE cells were cultured in the basal medium supplemented with 100 ng/mL noggin (Proteintech) and 10 µM SB431542 (Fujifilm Wako) for 4 days to differentiate into anterior foregut endoderm (AFE) cells. The AFE cells were cultured in the basal medium supplemented with 3 µM CHIR99021, 0.05 µM all-trans retinoic acid (Sigma-Aldrich) and 20 ng/mL BMP4 (Proteintech) for 4 days to differentiate into ventralized anterior foregut endoderm (VAFE) cells. For efficient distalization of VAFE cells, the cells were cultured in basal medium supplemented

with 3 μM CHIR99021, 10 ng/mL FGF10 (PeproTech), 10 ng/mL KGF (PeproTech) and 20 μM DAPT (Fujifilm Wako) for 7 days. After a 21-day differentiation step, NKX2-1$^+$ lung progenitor cells were isolated using FACS with mouse anti-human CPM antibody (1:100, Fujifilm Wako #014-27501) and Alexa Fluor 647 conjugated donkey anti-mouse IgG antibody (1:100, Thermo Fisher Scientific #A-31571).

**Induction of AT2 cells in FD-AOs**

FD-AOs were cultured in DCIK medium; Ham's F12 (Fujifilm Wako) containing 50 nM dexamethasone (Sigma-Aldrich), 100 μM 8-Br-cAMP (Biolog Life Science Institute), 100 μM 3-isobutyl-1-methylxanthine (Fujifilm Wako), 10 ng/mL KGF, 1% B-27 supplement, 0.25% BSA (Thermo Fisher Scientific), 15 mM HEPES (Thermo Fisher Scientific), 0.8 mM $CaCl_2$ (Fujifilm Wako), 0.1% ITS premix (Corning), and 50 U/mL P–S. Sorted 1 × $10^4$ CPM$^{high}$ cells were mixed with 5 × $10^5$ precultured HFLFs in 200 μL of 50% Growth Factor Reduced Matrigel (Corning) diluted with DCIK medium supplemented with 10 μM Y-27632. Mixed cells (200 μL) were placed on a 12-well cell culture insert (Corning), and 1 mL of DCIK medium containing 10 μM Y-27632 was added to the lower chamber. FD-AOs was cultured for 14 days; the medium in the lower chamber was replaced with DCIK medium every 2 to 3 days.

**Flow cytometry (FCM)**

Cell suspension, washing, and antibody dilution were performed with flow cytometry buffer; PBS containing 1% BSA and 10 μM Y-27632. Dissociated cells were fixed with 4% paraformaldehyde/PBS (Nacalai Tesque) and permeabilized with ice-cold methanol for intracellular staining. The following primary antibodies and staining reagents were used. Lyso Tracker Red DND-99 (1:10,000, Thermo Fisher Scientific #L7528), APC-conjugated mouse anti-human EpCAM antibody (1:100, Miltenyi Biotec Inc #130-113-260), rat anti-human podoplanin antibody (1:100, Thermo Fisher Scientific #16-9381-81), goat anti-human EpCAM antibody (1:100, Bio-Techne #AF960), mouse anti-mouse p21 antibody (1:25, Santa Cruz Biotechnology #sc-6246), mouse anti-human integrin β6 antibody (1:100, Bio-Techne #MAB4155), and purified mouse IgG2b isotype control antibody (1:100, BioLegend #402201). After being washed twice with flow cytometry buffer, the cells were stained with the following secondary antibodies. DyLight 550 conjugated donkey anti-rat IgG antibody (1:100, Thermo Fisher Scientific #SA5-10027), Alexa Fluor 647 conjugated donkey anti-goat IgG antibody (1:500, Thermo Fisher

Scientific #A-21447), Brilliant Violet 421 conjugated donkey anti-goat IgG antibody (1:100, Jackson Immuno Research #705-675-147), and Alexa Fluor 546 conjugated donkey anti-mouse 546 antibody (1:500, Thermo Fisher Scientific #A10036).

**qRT-PCR**

The PureLink RNA Mini Kit (Thermo Fisher Scientific) or RNeasy Micro Kit (Qiagen) was used to extract total RNA. Extracted total RNA was reverse-transcribed using ReverTra Ace qPCR RT Master Mix with gDNA Remover (Toyobo) according to the manufacturer's protocol. The primers used in this report are described in Tables S6 and S7. qPCR was performed using Power SYBR Green Master Mix (Thermo Fisher Scientific) or TaqPath qPCR Master Mix, CG (Thermo Fisher Scientific) on the StepOnePlus Real-Time PCR System (Thermo Fisher Scientific). The gene expression was normalized to the Eukaryotic 18S rRNA and compared with the human adult lung 5 donor pool (BioChain #R1234152-P, lot A811037) or each control. In compound screening using whole organoids of FD-AOs, the fold change relative to BLM-treated sample in each experiment was averaged and $\log_2$ transformed. The transformed value was visualized using the R package "gplots".

**Live cell imaging**

Images were obtained using a BZ-X710 microscope (Keyence). Images were jointed using BZ-X Analyzer (Keyence) and the whole matrix area was measured using the Image J software program (National Institutes of Health) (Schindelin et al., 2012). For quantification of the diameter and thickness of alveolar spheroids in each condition, 20 spheroids were randomly selected in each experiment and measured using BZ-X Analyzer.

**Isolation of epithelial cells and fibroblasts from FD-AOs**

Matrigel-embedded cells were carefully dissociated at 37 °C for 15 min via gentle pipetting with 0.1% Tripsin–EDTA (Thermo Fisher Scientific). Dissociated cells were stained with mouse anti-human EpCAM antibody (1:100, Santa Cruz Biotechnology #sc-66020). Magnetic-activated cell sorting (MACS) with goat anti-mouse IgG microbeads (1:5, Miltenyi Biotec Inc #130-048-401) was performed to separate epithelial cells ($EpCAM^+$) and fibroblasts ($EpCAM^-$) for the downstream analysis.

**RNA-seq**

The RNeasy Micro Kit was used to extract total RNA according to manufacturer's protocol. The RNA integrity number of each sample was confirmed to be over 8 using a 2100 BioAnalyer (Agilent Technologies). Sequence libraries of the fibroblast RNA were prepared using the NEBNext Ultra RNA LP Kit (New England Biolabs). For an analysis of GFP-SPC$^+$ cells, 10 ng of total RNA was reverse-transcribed and amplified with 8 cycles using SMART-Seq v4 Ultra Low Input RNA Kit for Sequencing (Clontech Laboratories). The amplified cDNAs were used to prepare sequence libraries using Nextera XT DNA Library Preparation Kit (Illumina). The library sequence was performed using 150 bp paired-end method by NovaSeq 6000 (Illumina).

**Bioinformatic analysis of RNA-seq data**

Sequenced reads of the fibroblasts were trimmed using fastp (Chen et al., 2018), and the trimmed reads were aligned to GRCh38 using STAR 2.7.3a (Dobin et al., 2013). Transcripts per million (TPM) values were calculated using RSEM (Li and Dewey, 2011). Sequencing reads of SPC-GFP$^+$ cells were aligned to GRCh37 using STAR 2.6.0c, and TPM values were calculated using Genedata Profiler Genome (Genedata). Low expression genes with average TPM values among the comparison data set less than 1 were excluded for downstream analyses. The principal component analysis (PCA) of $\log_2$ (TPM + 0.01) was performed using the R function "prcomp" and visualized using the R package "plotly". The R package DESeq2 (Love et al., 2014) was used for identification of Differentially Expressed Genes (DEGs). Volcano plots were visualized using the R package "ggplot2" and "ggrepel". Gene set enrichment analysis (GSEA) (Subramanian et al., 2005) was performed using the genes ordered by the *p*-values calculated by DESeq2. Enrichment analysis for gene ontology (GO) based on biological processes was performed using the PANTHER online software (Mi et al., 2021). A heatmap using $\log_2$ (TPM + 0.01) or $\log_2$ (fold change calculated by DESeq2) was visualized by the R package "gplots".

**Immunofluorescence analysis (IFA)**

FD-AOs were fixed with 4% paraformaldehyde/PBS. Blocking and antibody dilution were performed using blocking buffer (PBS containing 5% normal donkey serum [EMD-Millipore] and 1% BSA [Sigma-Aldrich]) unless otherwise stated. Paraffin-embedded specimens were sectioned into 3-μm slices, deparaffinized, and hydrated using the standard procedure. Antigen retrieval was performed with 10 mM sodium citrate (pH 6.0; Fujifilm Wako) at 98 °C for 30 min.

The retrieved sections were permeabilized with PBS containing 0.2% Triton X-100 (Nacalai Tesque), immersed in the blocking buffer, and stained with the following primary antibodies diluted with Can Get Signal Immunostain Enhancer Solution B (Toyobo): goat anti-human EpCAM antibody (1:100, Bio-Techne #AF960), rabbit anti-MYH11 antibody (1:100, Abcam #ab133567), goat anti-human RAGE antibody (1:100, Bio-Techne #AF1145), and rabbit anti-GFP antibody (1:200, Cell Signaling technology #2956). For cryosections (10 μm), fixed specimens were incubated in 30% sucrose (Nacalai Tesque) at 4 °C overnight, embedded in optimal cutting temperature compound (Sakura Finetek) and frozen at −70 °C. Cryosections were permeabilized with PBS containing 0.2% Triton X-100 or ice-cold methanol, blocked with blocking buffer, and stained with the following primary antibodies: goat anti-human SFN antibody (1:100, Abcam #ab77187) diluted with Can Get Signal immunostain Enhancer Solution B, mouse anti-human p53 antibody (1:100, Santa Cruz Biotechnology #sc-126), chicken anti-GFP antibody (1:500, Aves Labs #GFP-1020), mouse anti-mouse p21 antibody (1:25, Santa Cruz Biotechnology #sc-6246), goat anti-human EpCAM antibody (1:100, Bio-Techne #AF960), mouse anti-human EpCAM antibody (1:100, Santa Cruz Biotechnology #sc-66020), and goat anti-human RAGE antibody (1:100, Bio-Techne #AF1145). For secondary staining, Hoechst-33342 (1:1,000, Dojindo Molecular Technologies Inc.) and the following secondary antibodies were used: Alexa Fluor 647 conjugated donkey anti-goat IgG (1:500, Thermo Fisher Scientific #A-21447), Alexa Fluor 546 conjugated donkey anti-rabbit IgG (1:500, Thermo Fisher Scientific #A-10040), Alexa Fluor 488 conjugated donkey anti-rabbit IgG (1:500, Thermo Fisher Scientific #A-21206), Alexa Fluor 546 conjugated donkey anti-mouse IgG (1:500, Thermo Fisher Scientific #A-10036), and Alexa Fluor 488 conjugated donkey anti-chicken IgY (1:500, Jackson Immuno Research #703-485-155). Stained sections were embedded with ProLong Gold Antifade Mountant (Thermo Fisher Scientific) and imaged using a TCS SP8 confocal microscope (Leica Microsystems).

**Quantification of lineage marker-positive areas of epithelial cells in FD-AOs**

Images of cryosections obtained with a TCS SP8 confocal microscope were analyzed using the Image J software program. Binarized images were processed using the close function with 5 iterations, and debris smaller than 10 pixel units were eliminated. The SPC-GFP$^+$, AGER$^+$, and SPC-GFP$^+$AGER$^+$ area of EpCAM$^+$ area was measured, respectively.

**BLM treatment assay of FF-AOs**

Prior to BLM treatment, FF-AOs were prepared in DCIK medium supplemented with 10 μM Y-27632, 3 μM CHIR99021, and 10 μM SB431542. First, 2 × $10^5$ SPC-GFP$^+$ cells isolated from FD-AOs were suspended in 250 μL of the medium, seeded onto an Elplasia 96 well square bottom plate (Corning) coated with poly(2-hydroxyethyl methacrylate) (Sigma-Aldrich) and cultured for 2 days to form aggregates in each well. The aggregates were gently resuspended with 60 or 120 μL of Growth Factor Reduced Matrigel, and 20 μL of the suspension was dispensed into each well of a 12 well culture plate (60 μL for live cell imaging and RNA extraction; 120 μL for cell viability assay and flow cytometry). After the Matrigel solidified, 1 mL of the culture medium was added. The culture medium was replaced every 2 days until BLM treatment. Then, SPC-GFP$^+$ cell-derived FF-AOs were treated with 3 μg/mL BLM (Nippon Kayaku) from Day 6 to 9. BLM was washed out with PBS on Day 9, and the FF-AOs were cultured in dexamethasone-free DCIK medium without any of Y-27632, CHIR99021, or SB431542 from Day 9 to 12.

**Measurement of cell viability of FF-AOs.**

CellTiter-Glo 3D Cell Viability Assay (Promega) was used for measuring the viability of FF-AOs. FF-AOs were lysed with CellTiter-Glo 3D Cell Viability Assay reagent, transferred to a 96 well white clear bottom plate (Corning), and incubated for 30 min. Luminescence was measured using ARVO X5 (PerkinElmer).

**Transmission electron microscopy**

FD-AOs were fixed in 0.1M phosphate buffer (pH 7.4) containing 2.5% glutaraldehyde, 2% paraformaldehyde, 2% osmium tetroxide, 0.1% picric acid and 4% sucrose, as described previously (Gotoh *et al.*, 2014). En bloc staining was performed using 1% uranyl acetate. After the dehydration step, specimens were embedded in Epon 812 (Nacalai Tesque). Ultrathin sections were stained using uranyl acetate and lead citrate and examined by transmission electron microscopy (Hitachi #H-7650).

**LysoTracker staining for live cell imaging**

LysoTracker Red DND-99 (1:10,000) and Hoechst-33342 (1:1,000) were diluted with dexamethasone-free DCIK medium to prepare the staining solution. FF-AOs were stained with the staining solution and incubated for 1 h under 5% $CO_2$ at 37 °C. HBSS (+) (NacalaiTesque)

supplemented with 1%BSA was used for washing and imaging. Images were obtained using a BZ-X710 microscope.

**Measurement of SA-β-gal activity**

The 96-Well Cellular Senescence Assay (Cell Biolabs) was used for measuring SA-β-gal activity. The activity was measured according to manufacturer's protocol. The activity was corrected by the protein concentration measured by a BCA protein assay kit (Thermo Fisher Scientific).

**Measurement of MMP7 and TGFβ1 in the culture supernatant**

The culture medium in the lower chamber of FD-AOs on Day 17 was collected and centrifuged at 1,000 x g for 10 min. The retrieved supernatant was stored at −70 °C until measurement. ELISA Kits for MMP7 (Bio-Techne #DMP700) and TGFβ1 (Bio-Techne #DB100B) were used for quantification.

**Proteomic analysis**

FD-AOs were washed with PBS and were flash frozen in liquid nitrogen. Each sample was lysed with 100 µL of Lysis Buffer (10 mM Tris-HCl pH 8.0 [Cytiva], 7 M urea [Cytiva], 2 M thiourea [Fujifilm Wako], 5 mM magnesium acetate [Fujifilm Wako], and 4% [w/v] CHAPS [Dojindo Molecular Technologies Inc.] with Complete Protease Inhibitor Cocktail [Roche]). After sonication, each lysed sample was centrifuged at 20,000 x g for 30 min and the supernatant was collected. Approximately 100 µL of reducing buffer (10 mM DTT [Cytiva], 100 mM ammonium bicarbonate [Fujifilm Wako]) were added, and the mixture was incubated at 57 °C for 30 min. Next, 100 µL of alkylation buffer (50 mM iodoacetamide [Sigma-Aldrich] and 100 mM ammonium bicarbonate) were added, and the mixture was incubated for 30 min. Then, 100 µL of Sequencing Grade Modified Trypsin (Promega) and 100 µL of 50 mM ammonium bicarbonate were added, and the mixture was incubated at 30 °C for 16 h. The digested solution was dried in a centrifugal concentrator (TOMY #CC-105). Subsequently, 30 µL of 0.1% formic acid (Fujifilm Wako) was added and mixed by vortex. Each mixed sample was centrifuged at 20,000 x g for 10 min, and the supernatant was collected. Nanoscale liquid crystal–tandem mass spectrometric (nano LC–MS/MS) analysis was performed using the UltiMate 3000 liquid chromatograph (Thermo Fisher Scientific) and the Q-Exactive Plus mass spectrometer (Thermo Fisher Scientific). After a database search was conducted with the Mascot software program (Matrix Science), the results

were exported and analyzed with Scaffold (Proteome Software). A BLIB file was exported from Scaffold to obtain the database for data independent acquisition (DIA) analysis. For DIA analysis, nano LC–MS/MS measurement was performed twice on each sample. The acquired MS/MS data were annotated to human proteins by Uni-Prot (Consortium, 2019), and quantitative comparative analysis was performed using the Scaffold DIA software program (Proteome Software). Detected proteins with false discovery rate < 0.01 were analyzed, and the threshold for upregulation was set to $\log_2$ (fold change) > 0.4 ($n$ = 2 technical replicates). Pathway enrichment analysis of 427 proteins up-regulated by BLM treatment was conducted using the online Reactome software (Jassal et al., 2020).

**SUPPLEMENTAL REFERENCES**


Chen, S., Zhou, Y., Chen, Y., and Gu, J. (2018). fastp: an ultra-fast all-in-one FASTQ preprocessor. Bioinformatics *34*, i884-i890.

Consortium, U. (2019). UniProt: a worldwide hub of protein knowledge. Nucleic Acids Res *47*, D506-d515.

Coppé, J.P., Desprez, P.Y., Krtolica, A., and Campisi, J. (2010). The senescence-associated secretory phenotype: the dark side of tumor suppression. Annu Rev Pathol *5*, 99-118.

Dobin, A., Davis, C.A., Schlesinger, F., Drenkow, J., Zaleski, C., Jha, S., Batut, P., Chaisson, M., and Gingeras, T.R. (2013). STAR: ultrafast universal RNA-seq aligner. Bioinformatics *29*, 15-21.

Jassal, B., Matthews, L., Viteri, G., Gong, C., Lorente, P., Fabregat, A., Sidiropoulos, K., Cook, J., Gillespie, M., Haw, R., et al. (2020). The reactome pathway knowledgebase. Nucleic Acids Res *48*, D498-d503.

Kobayashi, Y., Tata, A., Konkimalla, A., Katsura, H., Lee, R.F., Ou, J., Banovich, N.E., Kropski, J.A., and Tata, P.R. (2020). Persistence of a regeneration-associated, transitional alveolar epithelial cell state in pulmonary fibrosis. Nat Cell Biol *22*, 934-946.

Li, B., and Dewey, C.N. (2011). RSEM: accurate transcript quantification from RNA-Seq data with or without a reference genome. BMC Bioinformatics *12*, 323.

Love, M.I., Huber, W., and Anders, S. (2014). Moderated estimation of fold change and dispersion for RNA-seq data with DESeq2. Genome Biol *15*, 550.

Mi, H., Ebert, D., Muruganujan, A., Mills, C., Albou, L.P., Mushayamaha, T., and Thomas, P.D. (2021). PANTHER version 16: a revised family classification, tree-based classification tool, enhancer regions and extensive API. Nucleic Acids Res *49*, D394-d403.

Neumark, N., Cosme, C., Jr., Rose, K.A., and Kaminski, N. (2020). The Idiopathic Pulmonary Fibrosis Cell Atlas. Am J Physiol Lung Cell Mol Physiol *319*, L887-l893.

Reyfman, P.A., Walter, J.M., Joshi, N., Anekalla, K.R., McQuattie-Pimentel, A.C., Chiu, S., Fernandez, R., Akbarpour, M., Chen, C.I., Ren, Z., et al. (2019). Single-Cell Transcriptomic Analysis of Human Lung Provides Insights into the Pathobiology of Pulmonary Fibrosis. Am J Respir Crit Care Med *199*, 1517-1536.

Rouillard, A.D., Gundersen, G.W., Fernandez, N.F., Wang, Z., Monteiro, C.D., McDermott, M.G., and Ma'ayan, A. (2016). The harmonizome: a collection of processed datasets gathered to serve and mine knowledge about genes and proteins. Database (Oxford) *2016*.

Subramanian, A., Tamayo, P., Mootha, V.K., Mukherjee, S., Ebert, B.L., Gillette, M.A., Paulovich, A., Pomeroy, S.L., Golub, T.R., Lander, E.S., et al. (2005). Gene set enrichment analysis: a knowledge-based approach for interpreting genome-wide expression profiles. Proc Natl Acad Sci U S A *102*, 15545-15550.

Yamamoto, Y., Gotoh, S., Korogi, Y., Seki, M., Konishi, S., Ikeo, S., Sone, N., Nagasaki, T., Matsumoto, H., Muro, S., et al. (2017). Long-term expansion of alveolar stem cells derived from human iPS cells in organoids. Nat Methods *14*, 1097-1106.

Zepp, J.A., Morley, M.P., Loebel, C., Kremp, M.M., Chaudhry, F.N., Basil, M.C., Leach, J.P., Liberti, D.C., Niethamer, T.K., Ying, Y., et al. (2021). Genomic, epigenomic, and biophysical cues controlling the emergence of the lung alveolus. Science *371*.
